# Independent-effect comparison of five crosslinking procedures for Progressive Keratoconus based on Keratometry and the ABCD Grading System using Generalized Estimating Equations (GEE)

**DOI:** 10.1186/s12886-022-02744-w

**Published:** 2023-01-10

**Authors:** Yu Liu, Dan Shen, Hao-yu Wang, Deng-feng Liang, Qing-yan Zeng

**Affiliations:** 1 Aier School of Ophthalmology, Central South University, Changsha, China; 2 Hankou Aier Eye Hospital, Wuhan, China; 3 Aier Cornea Institute, Beijing, China; 4 Wuhan University, Wuhan, China; 5 Hubei University of Science and Technology, Xianning, China

**Keywords:** Crosslinking, GEE, Keratoconus, PTK, ABCD Grading System

## Abstract

**Purpose:**

Corneal collagen crosslinking (CXL) is an effective treatment for progressive keratoconus. Multiple CXL modalities are clinically available. The present study compared the 1 year outcomes of five types of CXL procedures for progressive keratoconus in a Chinese population using generalized estimating equations (GEE).

**Methods:**

This retrospective study included 239 eyes in 171 patients with keratoconus who underwent CXL and were followed up for 1 year. Five CXL procedures were assessed, including *Accelerated Transepithelial CXL*, *Iontophoresis CXL* for 10 min, CXL plus phototherapeutic keratectomy (*CXL-plus-PTK*), *High-Fluence Accelerated CXL*, and *Accelerated CXL*. Patients treated with the *Accelerated CXL* procedure represented the reference group. Primary outcomes were visual acuity change, spherical equivalence, endothelial cell density, mean keratometry (K_mean_), maximum keratometry (K_max_), minimum corneal thickness (MCT), and the ABCD Grading System, consisting of **A** (staging index for ARC; ARC = anterior radius of curvature), **B** (staging index for PRC, PRC = posterior radius of curvature), and **C** (staging index for MCT) values 1 year postoperatively compared to baseline. Secondary outcomes were corrected GEE comparisons from each procedure versus the *Accelerated CXL* group.

**Results:**

The *Accelerated Transepithelial CXL* group had lower performance than the *Accelerated CXL* group according to K_mean_ and K_max_. The *CXL-plus-PTK* group performed significantly better than the reference group as reflected by K_max_ (β = -0.935, *P* = 0.03). However, the *CXL-plus-PTK* group did not perform as well for **B** and **C**, and the *Iontophoresis CXL* group performed better for **C**.

**Conclusions:**

The *CXL-plus-PTK* procedure was more effective than the *Accelerated CXL* procedure based on K_max_, and the *Iontophoresis CXL* procedure performed better on the **C** value based on the ABCD Grading System.

**Supplementary Information:**

The online version contains supplementary material available at 10.1186/s12886-022-02744-w.

## Introduction

Corneal collagen crosslinking (CXL) is an effective treatment modality for progressive keratoconus and other corneal diseases [1]. The corneal stroma is primarily comprised of regularly arranged collagen fibers and their interconnections. In patients with keratoconus, the interconnections are impaired, decreasing corneal mechanical strength. Wollensak et al. [2] demonstrated for the first time that CXL increases corneal mechanical strength in keratoconus. This procedure involves photochemical reactions on the surface of the collagen fibers and in the protein networks surrounding the collagen [3]. In addition, CXL increases collagen fiber diameters and improves resistance of the corneal stroma against multiple degrading enzymes.

These findings support the use of photochemical treatments in CXL to increase corneal strength and prevent or delay keratoconus progression. The most commonly used method in clinical practice is the standard Dresden protocol for corneal crosslinking (S-CXL). However, S-CXL is time-consuming, and although prior studies report comparable efficacies of accelerated CXL (A-CXL) and S-CXL [4, 5], some clinical centers do not currently use conventional S-CXL to treat keratoconus. Moreover, removal of the corneal epithelium may cause pain, discomfort, temporary vision reduction, and decreased corneal clarity (haze), and can increase the risk of infectious keratitis [6–8]. Many patients cannot undergo epithelium-off (epi-off) surgery due to insufficient corneal thickness (< 400 μm). Nevertheless, retaining the corneal epithelium (epi-on surgery) could decrease the efficacy of CXL as the epithelium can impede the penetration of ultraviolet (UV) radiation and riboflavin. Recently, multiple studies have reported means to improve CXL treatment procedures, such as immersing riboflavin into the stroma by various means, increasing UV irradiation energy and using an excimer laser rather than mechanics to remove the epithelium. Few randomized controlled trials have evaluated the relative efficacies of multiple CXL modalities. In available trials, the sample size or the treatment protocol types are limited, and inclusion criteria, such as ethnographics, age group, and baseline data, vary [9], precluding definitive recommendations of CXL procedure types for patients with progressive keratoconus. For example, previous studies have demonstrated that pediatric keratoconus is more aggressive than adult keratoconus, and age is an important influence on the efficacy of CXL [10, 11]. A study using a generalized estimating equation (GEE) could be used to compare the efficacy of different types of CXL procedures with varying combinations of riboflavin and irradiation power for treatment of progressive keratoconus in pediatric patients, as the generalized linear model correction could analyze outcomes to exclude the effects of sex, age, baseline data heterogeneity, and bilateral surgery [12].

Currently, according to the Global Consensus on Keratoconus and Ectatic Diseases (2015) [13], many investigators concur that a more comprehensive evaluation system should be used to evaluate keratoconus progression, rather than simply the maximum keratometry (K_max_) of the corneal anterior surface. The ABCD Grading System [14] uses the anterior (**A**) and posterior (B) radiuses of curvature at the cone area, corneal thickness at the thinnest region of the cornea (**C**), and best corrected distance vision (**D**). Recent studies have identified that more than half of patients demonstrate quantifiable progress using the ABCD Grading System earlier than with measurement of K_max_change alone [15], pointing to the utility of a combined progression system to track progress following CXL [16].

The present study compared the independent effects of five CXL procedures for progressive keratoconus at 1 postoperative year with follow-up based on keratometry and the ABCD Grading System.

## Materials and methods

### Data set and study design

A retrospective medical chart review was conducted on all consecutive patients with progressive keratoconus at the Hankou Aier Eye Hospital (Wuhan, Hubei province, China) who underwent CXL treatment between July 7, 2014 and August 22, 2021. Patients who returned for a follow-up visit after 1 year were included in the study. All patients provided written informed consent prior to surgery, and surgeries were performed by the same operator (Q. Y. Zeng).

An increase of at least 1 diopter (D) in maximum keratometry (K_max_) derived from computerized corneal topography during the preceding 12 months was required for inclusion. Patients with previous refractive surgeries or corneal history of ocular surface or other eye disorders were excluded. In addition, patients whose data could not be reviewed for any reason were classified as being lost to follow-up and excluded from the study.

### Surgical technique

Patients were included regardless of treatment protocols. In total, five different treatment combinations were included in the study: *Accelerated Transepithelial CXL*, *Iontophoresis CXL* for 10 min, CXL plus phototherapeutic keratectomy (*CXL–plus-PTK*), *High-Fluence Accelerated CXL*, and *Accelerated CXL*. When the thinnest corneal thickness of the eye was < 450 μm, patients were randomized to undergo either *Accelerated Transepithelial CXL* or *Iontophoresis CXL*. When the thinnest corneal thickness of the eye was ≥ 450 μm, the patients or their guardians elected whether to undergo the *CXL-plus-PTK* procedure to correct the irregularity of the epithelium. If preferred, the patients underwent the *CXL-plus-PTK* procedure. If the patients refused, they were randomly selected to receive the *High-Fluence Accelerated CXL* or *Accelerated CXL* procedure.

1. *Accelerated Transepithelial CXL*: In the first step, 0.25% riboflavin (Paracel Part I, Avedro Inc., USA) containing 0.02% benzalkonium chloride (BAC) and 0.85% hydroxypropyl methyl cellulose (HPMC) was applied onto the cornea every 90 s for 4 min. Thereafter, part I solution was rinsed with 0.22% riboflavin (Paracel Part II, Avedro), and part II solution was instilled every 90 s over the next 6 min. UV-A was applied using the Avedro KXL System (Avedro Inc., Waltham, USA) with 30 mW/cm^2^ UV power for 8 min with a 1 s on/off cycle (7.2 J/cm^2^).
2. *Iontophoresis CXL*: Patients underwent iontophresis for stromal imbibition, with 0.35 ml 0.1% Ricrolin + riboflavin solution (Sooft, Montegiorgio, Italy) applied to 0.8 mm of the cornea with a suction ring and delivered by an electric generator I-ON CXL (Sooft) set at 1 mA through inox electrodes for 10 min. The treated cornea was subsequently exposed to UV-A light (Vega, CSO, Firenze, Italy) for 9 min at an irradiance of 10 mW/cm^2^ (5.4 J/cm^2^).
3. *CXL-plus-PTK*: The corneal epithelium was ablated in a 7 mm zone with an intended depth of 50 μm using an excimer laser (Schwind eye-tech-solutions GmbH & Co. KG, Kleinostheim, Germany). CXL was then performed with 0.1% dextran-free riboflavin (VibeX Rapid, Avedro) instilled every 90 s for 10 min. Subsequntly, it was placed under UA irradiation for 4 min at 30 mW/cm^2^ (7.2 J/cm^2^, Avedro).
4. *High-Fluence Accelerated CXL*: The corneal epithelium was removed with a blunt knife in a 10 mm zone. Riboflavin and UV irradiation were used as described in the CXL-plus-PTK group (7.2 J/cm^2^).
5. *Accelerated CXL*: The epithelium was also removed by a blunt knife in a 10 mm zone. Riboflavin used for A-CXL was comprised of riboflavin 0.1% and dextran 20.0% (Ricrolin, Sooft). UV irradiation was used as described in the I-CXL group (5.4 J/cm^2^).

The operator verified irradiance prior to each treatment. The five CXL procedures are summarized in Table 1.

**Table 1:** Five Crosslinking Treatment Procedures

**Parameter**	**Treatment Procedures**				
	**Accelerated Transepithelial CXL**	**Iontophoresis CXL**	**CXL-plus-PTK**	**High-Fluence Accelerated CXL**	**Accelerated CXL**
Fluence (total)(J/cm^2^)	7.2	5.4	7.2	7.2	5.4
Soak time and interval (minutes)	10 (1.5)	10	10 (1.5)	10 (1.5)	30 (2)
Intensity(mW/cm^2^)	30	9	30	30	10
Treatment time(minutes)	8	10	4	4	9
Irradiation mode (interval)	Pulsed(1 sec on-1 sec off)	Continuous	Continuous	Continuous	Continuous
Epithelium status	On	On	Off	Off	Off
De-epithelialization method	/	/	PTK	Mechanical	Mechanical
Riboflavin	ParaCel	Ricrolin+	Vibex Rapid	Vibex Rapid	Ricrolin
Riboflavin osmolarity	Iso-	Iso-	Iso-	Iso-	Iso-
Light source	KXL	VEGA	KXL	KXL	VEGA

*CXL *corneal crosslinking

### Pain medication and postoperative care

All patients received 0.5% levofloxacin drops four times daily for 3 days prior to surgery. Thirty minutes before surgery, patients received 2% pilocarpine (Sigma-Aldrich, St. Louis, MO, USA) and 0.4% oxybuprocaine hydrochloride (Bausch & Lomb Pty Ltd., NSW, Australia) drops three times, with 5 min between administrations.

At the end of the surgery, the corneal surface was dressed with a therapeutic soft contact lens (Bausch & Lomb Pty Ltd.) for at least 24 h until the epithelium was completely healed.

### Assessments

Contact lens wearers were instructed to discontinue use for a minimum of 3 weeks prior to the preoperative eye examination. During the baseline visit and postoperative visits at 1, 3, 6, and 12 months, the following assessments were performed: uncorrected distance visual acuity (UDVA; in logarithm of the minimum angle of resolution [LogMAR] units), corrected distance visual acuity (CDVA; in LogMAR units), and spherical equivalence (SE); mean keratometry (K_mean_), maximum keratometry (K_max_), minimum corneal thickness (MCT), **A** (staging index for ARC; ARC = anterior radius of curvature), **B** (staging index for PRC, PRC = posterior radius of curvature), and **C** (staging index for MCT, measured with an Oculus Pentacam; Oculus, Wetzlar, Germany). Confocal microscopy was performed using an HRT3 microscope (Heidelberg Engineering, Heidelberg, Germany) to measure endothelial cell density (ECD). Adverse events were defined as any documented medical complication, including keratitis or corneal ulceration, or retreatment at any time point within 1 year after CXL.

### Statistical analysis

A multivariable linear regression model with GEE correction was used to correct for cases in which a patient underwent bilateral CXL. The *Accelerated CXL* group was the reference group, and the other four procedures were compared against this group. Further, to eliminate the effect of age on the determined variable, patients were divided into two groups depending on age (< 18 years or ≥ 18 years). All secondary outcomes were analyzed using GEE correction.

Baseline measurement normality was assessed using Q-Q plots. Categorical variables were presented as numbers and percentages. A one-way analysis of variance was used to analyze differences in baseline characteristics among the five groups. To account for multiple comparisons, a Gabriel post hoc test was performed. A paired Student’s t-test statistical analysis was performed to compare the change of clinical parameters between the baseline and 1 year follow-up time points in each group. Variables were assessed for multicollinearity. All statistical analyses were performed with SPSS version 25.0 (IBM, Armonk, NY, USA). A *P*-value < 0.05 was considered statistically significant.

## Results

### Patient characteristics

Data from 368 eyes in 266 patients diagnosed with progressive keratoconus who underwent CXL treatment were recorded. Fifty patients (68 eyes) were reviewed at the local hospital instead of our center after their condition stabilized. Forty-five patients (61 eyes) did not complete the 1 year follow-up and were excluded. Finally, the study included 239 eyes from 171 patients. The mean patient age was 20.67 ± 6.02 years (range: 6 to 46 years). Of the 171 patients, 126 (73.68%) were male, and 60 (35.09%) were younger than 18 years. Demographic and baseline data are summarized in Table 2.

**Table 2:** Demographic and Baseline Data of Five Groups

**Baseline Parameter**	**Treatment Procedures**				
	**Epi-on**		**Epi-off**		
	**Accelerated Transepithelial CXL **	**Iontophoresis CXL**	**CXL-plus-PTK**	**High-Fluence Accelerated CXL**	**Accelerated CXL**
Age, y(mean, range)	22 (10-37)	20.47 (11-39)	22.07 (15-39)	18.39 (10-35)	21.08 (6-43)
Pediatric, n (%)	10 (31.25%)	18 (28.13%)	13 (39.39%)	16 (51.61%)	10 (38.46%)
Male, n (%)	22 (68.75%)	46 (71.88%)	29 (67.44%)	27 (87.1%)	18 (59.23%)
Laterality right, n (%)	21 (50%)	34 (45.33%)	16 (34.04%)	21 (51.22%)	18 (52.94%)
UDVA (LogMAR)	0.66 ± 0.29	0.82 ± 0.36	0.77 ± 0.37	0.73 ± 0.38	0.82 ± 0.43
CDVA (LogMAR)	0.28 ± 0.17	0.31 ± 0.25	0.26 ± 0.19	0.18 ± 0.17	0.24 ± 0.23
SE (D)	-7.05 ± 4.88	-7.62 ± 4.72	-6.72 ± 3.75	-4.99 ± 2.55	-5.47 ± 2.99
K_mean_ (D)	49.90 ± 4.47	50.59 ± 5.27	47.37 ± 3.48	46.75 ± 3.79	47.06 ± 2.73
K_max_ (D)	59.97 ± 8.41	60.31 ± 9.41	55.25 ± 7.15	53.02 ± 7.91	54.60 ± 5.68
MCT (minimum, μm)	436 ± 34 (374)	438 ± 34 (359)	481 ± 24 (451)	498 ± 32 (455)	486 ± 29 (450)
A	3.49 ± 2.34	3.76 ± 2.74	2.33 ± 1.66	2.03 ± 1.95	2.30 ± 1.38
B	5.09 ± 2.71	5.37 ± 3.09	3.76 ± 2.38	2.98 ± 2.26	3.65 ± 1.75
C	2.32 ± 0.85	2.17 ± 0.68	1.30 ± 0.47	1.04 ± 0.54	1.23 ± 0.48
ECD (cells/mm^2^)	2678 ± 249	2632 ± 245	2701 ± 276	2582 ± 310	2621 ± 289

*CXL* corneal crosslinking, *UDVA* uncorrected distance visual acuity, *LogMAR* logarithm of the minimum angle of resolution, *CDVA* corrected distance visual acuity, *SE* spherical equivalence, *D* diopters, *K*_*mean*_ mean keratometry, *K*_*max*_ maximum keratometry, *MCT* minimum corneal thickness, *ARC* anterior radius of curvature, *A* staging index for ARC, *PRC* posterior radius of curvature, *B* staging index for PRC, *C* staging index for MCT, *ECD* endothelial cell density

### Primary outcome: results without GEE correction

In analysis of the 239 eyes regardless of procedure type, significant improvements were observed in visual acuity, K_mean_, K_max_, and A value 1 year after surgery. UDVA improved by 0.08 ± 0.25 LogMAR (*P* < 0.001), and CDVA improved by 0.06 ± 0.17 LogMAR (*P* < 0.001). K_mean_ decreased significantly by 0.19 ± 1.18 (*P* = 0.005). K_max_ decreased by 1.03 ± 2.45 D at 1 year postoperatively (*P* < 0.001), and 153 eyes (64.02%) exhibited a downward trend in K_max_ at the 1 year follow-up (< 0 D change). The **A** value significantly improved at 1 year postoperatively, as demonstrated by decreases of 0.16 ± 0.81 (*P* = 0.002). The change of B value was not statistically significant (*P* = 0.21). MCT decreased significantly by 4.87 ± 17.36 μm (*P* < 0.001), and the **C** value increased significantly by 0.08 ± 0.38 (*P* = 0.002). SE improved by 0.52 ± 2.82 D at 1 year postoperatively (*P* = 0.005), and ECD was unchanged.

**Fig. 1:**
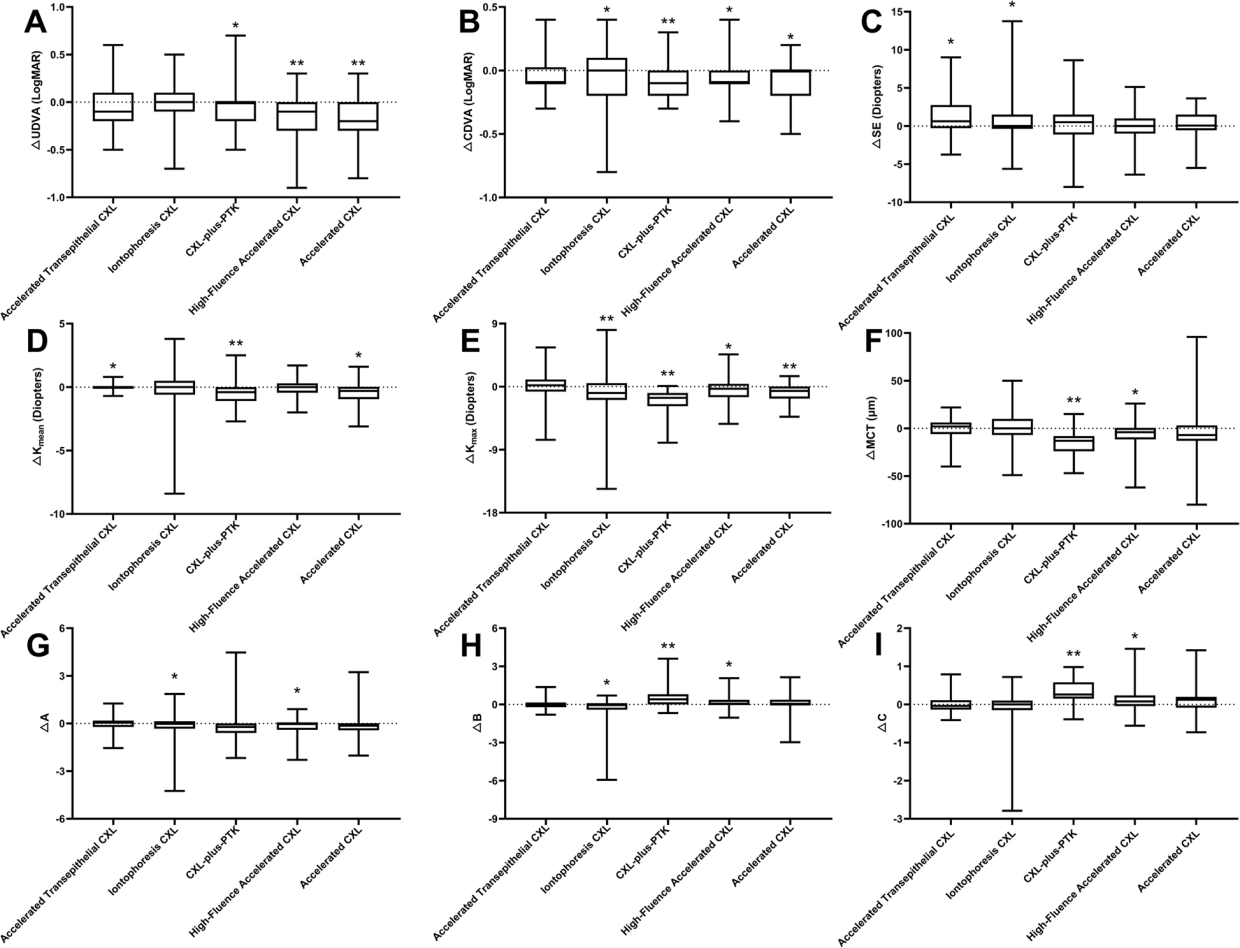
Box and whiskers graphs for changes of (**A**) uncorrected distance visual acuity (UDVA) (LogMAR = logarithm of the minimum angle of resolution), **B** corrected distance visual acuity (CDVA; LogMAR), (**C**) spherical equivalence (SE) (D = diopters) (**D**) mean keratometry (K_mean_; D), (**E**) maximum keratometry (Kmax; D), (**F**) minimum corneal thickness(MCT; μm), (**G**) **A** (staging index for ARC; ARC = anterior rcadius of curvature), (**H**) **B** (staging index for PRC, PRC = posterior radius of curvature), and (**I**) C (staging index for MCT) after five different corneal cross-linking (CXL) procedures with progressive keratoconus at 1 year postoperatively. △ = change compare 1-year value to baseline, * indicates *P* < 0.05 statistically significant difference compared to baseline, ** indicates *P* < 0.001 statistically significant difference compared to baseline

The average changes in each parameter 1 year after the five CXL procedure types are shown in Fig. 1 and Supplemental Digital Content 1. With the exception of the *Accelerated Transepithelial CXL* group, K_max_ was significantly decreased in all groups, and the change was largest in the *CXL-plus-PTK* group (*P* < 0.001). All five procedures stabilized the progress of keratoconus.

According to the ABCD Grading System, **A** and **B** values were significantly decreased in the *Iontophoresis CXL* group at 1 year postoperatively. The **A** value was significantly decreased in the *High-Fluence Accelerated CXL* group. However, the **B** and **C** values significantly increased in the *High-Fluence Accelerated CXL* and *CXL-plus-PTK* groups.

### Secondary outcomes: comparison of crosslinking procedures with GEE correction

#### Visual acuity and refractive parameters

Compared with the reference group, UDVA, CDVA, and SE were unchanged by treatment procedures (Supplemental Digital Content 2).

#### Corneal topography

After GEE correction, the *Accelerated Transepithelial CXL* and *Iontophoresis CXL* groups did not perform as well as the reference group (*Accelerated CXL*) according to K_mean_ (β = 1.094, *P* = 0.000; and β = 0.548, *P* = 0.006). The *Accelerated Transepithelial CXL* group also had decreased performance relative to the reference group according to K_max_ (β = 2.020, *P* = 0.001). Only the *CXL-plus-PTK* group differed significantly in reduction of the K_max_ compared with the reference group (β = -0.935, *P* = 0.03). According to MCT, the *CXL-plus-PTK* group did not perform as well as the reference group after GEE were corrected for baseline differences (β = -11.208, *P* = 0.009). The GEE analyses performed for K_mean_, K_max_, and MCT are showed in Table 3.

**Table 3:** Secondary Outcomes: GEEs Analysis for Changes of K_mean_, K_max_, and MCT after Five Corneal Crosslinking Procedures at 12 Months Postoperatively

**Parameter**	**ΔK**_**mean**_**(D)**			**ΔK**_**max**_ **(D)**			**△MCT(μm)**		
	**β-coefficient**^**a**^	**95% CI**	***P*-value**^**b**^	**β- coefficient**^**a**^	**95% CI**	***P*-value**^**b**^	**β-coefficient**^**a**^	**95% CI**	***P*-value**^**b**^
Accelerated Transepithelial CXL	1.094	0.568 to1.619	**0.000**	2.020	0.865 to 3.176	**0.001**	1.683	-8.415 to 11.781	0.744
Iontophoresis CXL	0.548	0.154 to 0.942	**0.006**	0.685	-0.207 to 1.577	0.132	3.036	-5.691 to 11.763	0.495
CXL-plus-PTK	-0.079	-0.497 to 0.339	0.712	-0.935	-1.778 to -0.092	**0.030**	-11.208	-19.615 to -2.801	**0.009**
High-Fluence Accelerated CXL	0.351	-0.006 to 0.708	0.054	0.346	-0.492 to 1.185	0.418	1.926	-7.792 to 11.644	0.698
Age＜18 y	-0.024	-0.318 to 0.270	0.875	-0.272	-0.864 to 0.321	0.369	-5.226	-9.735 to -0.717	**0.023**
Age ≥18 y	0								
UDVA (LogMAR)	-0.200	-0.863 to 0.463	0.555	-0.611	-1.841 to 0.620	0.331	-1.544	-10.206 to 7.118	0.727
CDVA (LogMAR)	0.151	-0.842 to 1.144	0.765	0.659	-1.301 to 2.619	0.510	3.960	-6.302 to 14.222	0.449
SE (D)	-0.002	-0.054 to 0.049	0.929	0.046	-0.053 to 0.145	0.359	-0.314	-0.921 to 0.293	0.311
K_mean_ (D)	-0.078	-0.169 to 0.014	0.096	-0.008	-0.193 to 0.177	0.935	0.145	-1.306 to 1.595	0.845
K_max_ (D)	0.046	-0.010 to 0.103	0.106	-0.107	-0.207 to -0.007	**0.036**	-0.194	-0.990 to 0.603	0.634
MCT (μm)	-0.003	-0.008 to 0.002	0.229	-0.018	-0.034 to -0.003	**0.020**	-0.209	-0.399 to -0.019	**0.031**
A	0.044	-0.188 to 0.275	0.711	0.410	-0.036 to 0.856	0.072	-0.156	-2.944 to 2.631	0.912
B	-0.049	-0.207 to 0.109	0.543	-0.173	-0.465 to 0.119	0.246	-0.656	-2.569 to 1.258	0.502
C	-0.435	-0.718 to -0.151	**0.003**	-1.173	-1.888 to -0.459	**0.001**	-5.584	-14.218 to 3.049	0.205

*GEEs* generalized estimating equations, *CXL* corneal crosslinking, Bold entries are statistically significant (*P* ＜ 0.05), Each treatment procedures was compared to the reference group (Accelerated CXL), *Δ* difference between 12 months post-CXL and pre-CXL, *95% CI* 95% confidence interval, *PTK* phototherapeutic keratectomy, *UDVA* uncorrected distance visual acuity, *LogMAR* logarithm of the minimum angle of resolution, *CDVA* corrected distance visual acuity, *SE* spherical equivalence, *D* diopters, *K*_mean_ mean keratometry, *Kmax* maximum keratometry, *MCT* minimum corneal thickness, *ARC* anterior radius of curvature, *A* staging index for ARC, *PRC* posterior radius of curvature, *B* staging index for PRC, *C* staging index for MCT

^a^The **β-coefficient** refers to how a dependent variable will change per unit increase in the predictor variable

^b^**P-value** from generalized estimating equations, corrected for baseline

Our secondary analysis for the ABCD Grading System (**A**, **B**, **C**) revealed that the *Accelerated Transepithelial CXL* group had decreased performance relative to the reference group in the **A** value (β = 0.372, *P* = 0.036). The *CXL-plus-PTK* group did not perform as well as the reference group according to the **B** value (β = 0.421, *P* = 0.01) and **C** value (β = 0.237, *P* < 0.001). The *Iontophoresis CXL* group performed significantly better than the reference group according to the **C** value (β = -0.136, *P* = 0.034) (Table 4).

**Table 4:** Secondary Outcomes: GEEs Analysis for Changes of A, B, and C after Five Corneal Crosslinking Procedures at 12 Months Postoperatively

**Parameter**	**△ A**			**△ B**			**△ C**		
	**β-coefficient**^**a**^	**95% CI**	***P*-value**^**b**^	**β-coefficient**^**a**^	**95% CI**	***P*-value**^**b**^	**β-coefficient**^**a**^	**95% CI**	***P*-value**^**b**^
Accelerated Transepithelial CXL	0.372	0.024 to 0.719	**0.036**	0.008	-0.309 to 0.326	0.958	-0.035	-0.190 to 0.120	0.658
Iontophoresis CXL	0.141	-0.160 to 0.443	0.359	-0.218	-0.472 to 0.036	0.093	-0.136	-0.262 to -0.010	**0.034**
CXL-plus-PTK	0.023	-0.370 to 0.324	0.897	0.421	0.102 to 0.740	**0.010**	0.237	0.111 to 0.363	**0.000**
High-Fluence Accelerated CXL	-0.010	-0.267 to 0.286	0.944	0.046	-0.232 to 0.324	0.745	-0.001	-0.164 to 0.163	0.995
Age＜18 y	-0.177	-0.364 to 0.009	0.063	-0.030	-0.127 to 0.187	0.709	0.095	-0.005 to 0.185	**0.039**
Age ≥18 y	0								
UDVA (LogMAR)	0.010	-0.351 to 0.372	0.966	-0.181	-0.663 to 0.301	0.463	-0.030	-0.245 to 0.184	0.782
CDVA (LogMAR)	-0.310	-0.910 to 0.291	0.312	-0.234	-0.651 to 0.184	0.273	-0.195	-0.422 to 0.032	0.093
SE (D)	0.015	-0.023 to 0.053	0.441	0.005	-0.020 to 0.030	0.700	0.004	-0.008 to 0.016	0.551
K_mean_ (D)	0.015	-0.069 to 0.100	0.724	0.009	-0.059 to 0.076	0.801	0.009	-0.021 to 0.040	0.541
K_max_ (D)	0.073	0.024 to 0.122	0.003	0.036	-0.003 to 0.076	0.071	0.007	-0.008 to 0.023	0.354
MCT (μm)	0.000	-0.006 to 0.005	0.875	-0.002	-0.006 to 0.002	0.362	-0.003	-0.008 to 0.003	0.365
A	-0.303	-0.518 to -0.089	**0.006**	-0.037	-0.178 to 0.104	0.607	0.011	-0.049 to 0.072	0.714
B	0.000	-0.098 to 0.097	0.992	-0.078	-0.175 to 0.019	0.117	-0.022	-0.076 to 0.032	0.430
C	-0.161	-0.454 to 0.132	0.281	-0.191	-0.432 to 0.049	0.118	-0.196	-0.513 to 0.122	0.227

*GEEs *generalized estimating equations,* CXL *corneal crosslinking,Bold entries are statistically significant (*P *＜ 0.05),Each treatment procedures was compared to the reference group (Accelerated CXL),* Δ *difference between 12 months post-CXL and pre-CXL,* 95% CI  *95% confidence interval,* PTK *phototherapeutic keratectomy,* UDVA *uncorrected distance visual acuity,* LogMAR *logarithm of the minimum angle of resolution,* CDVA *corrected distance visual acuity,* SE *spherical equivalence, *D* diopters, *K*_*mean*_ mean keratometry, *Kmax* maximum keratometry,* MCT *minimum corneal thickness,* ARC *anterior radius of curvature,* A *staging index for ARC,* PRC *posterior radius of curvature,* B *staging index for PRC,* C *staging index for MCT

^a^The **β-coefficient** refers to how a dependent variable will change per unit increase in the predictor variable

^b^**P-value** from generalized estimating equations, corrected for baseline

#### Age group analyses

According to age group analyses of GEE correction, pediatric patients (< 18 years) exhibited a more significant reduction in MCT (β = -5.226, *P* = 0.023) and **C** value changes (β = 0.095, *P* = 0.039) than adults. Similar results were obtained for other parameters.

### Progression and retreatment

According to the most quoted definition of progression (K_max_increase > 1.0 D or CDVA decrease ≥ 2 lines in 1 year) [17], 26 eyes (10.88%) showed progression, with nine eyes (21.43%) in the *Accelerated Transepithelial CXL* group, ten eyes (13.33%) in the *Iontophoresis CXL* group, zero eyes in the *CXL-plus-PTK* group, four eyes (9.76%) in the *High-Fluence Accelerated CXL* and three eyes (8.82%) in the *Accelerated CXL* group. Most of the eyes (19) were in the epi-on groups. However, with extended follow-up time, and when the overall situation of the patients was considered, ten eyes (4.18%) underwent retreatment by the final follow-up of November 10, 2022. All of these cases were in the epi-on CXL groups.

### Adverse events

Opacity of the corneal stroma at the central or paracentral areas occurred in five eyes (2.09%) of three patients during the follow-up. All three patients with stromal opacity underwent *High-Fluence Accelerated CXL*. Patient ages were 10, 11, and 15 years. The minimum stromal thickness after epithelial removal ranged from 411 to 429 μm of the five eyes. Among the three pediatric patients, two patients (four eyes) had a history of sunlight exposure early in the postoperative period. After treatment with 0.1% fluorometholone (Allergan, Irvine, CA) and corneal protection to avoid direct irritation from sunlight, corneal transparency was restored in two eyes, and corneal opacity was decreased in three eyes (Supplementary Figure). No infections or other adverse events were observed in slit-lamp examination.

## Discussion

To our knowledge, this is the first retrospective longitudinal study to compare the independent effects of different CXL procedures in treatment of keratoconus based on keratometry and the ABCD Grading System. All five procedure types stabilized disease, and improved visual acuity and keratometry values at 1 year postoperatively, according to findings without GEE corrections (Fig. 1 and Supplemental Digital Content 1). Secondary analysis revealed that no treatment procedure resulted in significant differences in UDVA, CDVA, and SE compared with the *Accelerated CXL*group. These findings were consistent with some previous reports [18–20]. Contrastingly, some studies have reported that different CXL procedures result in significantly improved visual acuity [21, 22]. All of these results are influenced by interaction effects of various factors. Therefore, we used the GEE to correct for the effects of age, baseline data heterogeneity, and bilateral surgery on treatment outcomes.

The UV-A irradiation energy used for the *Accelerated Transepithelial CXL* procedure was 7.2 J/cm^2^, which was higher than that of the *Accelerated CXL* procedure (5.4 J/cm^2^). It was less effective, as defined by K_mean_, K_max_, and Avalue at 1 year postoperatively. This could be because the corneal epithelium block the penetration of riboflavin and ultraviolet irradiation. Increasing UV-A irradiation energy could potentially improve the efficacy of CXL treatment, and benzalkonium chloride could disrupt the tight junctions of the corneal epithelium [23]. A prior study used the conventional UV-A energy (5.4 J/cm^2^) for *Accelerated Transepithelial CXL*and reported the same trend of kerametory changes not only at 1 year but also at 6, 18, and 24 months postoperatively [24]. The *Accelerated Transepithelial CXL* procedure had the lowest efficacy among the five CXL procedures 1 year postoperatively, as measured by K_mean_ and K_max_ values.

Our findings suggested that the *CXL-plus-PTK* procedure was the most effective based on reduction in K_max_value. Excimer laser ablation combined with CXL treatment has been proposed as an ideal technique due to its optimal refractive outcome that can provide both stability and functional vision, by both stabilizing and reshaping the cornea [25]. Previous studies also suggested that the *CXL-plus-PTK*procedure is more effective than the S-CXL procedure [26, 27]. Further, the improved efficacy could last up to 2 years after surgery [28]. Ozge et al. reported no obvious differences between the *CXL-plus-PTK* and S-CXL groups until the third postoperative year, although the corneal epithelium was removed in both procedures. One possible reason for the improved efficacy of the *CXL-plus-PTK *procedure is that PTK could ablate part of the Bowman layer and the irregular stroma on top of the cone area. Ablation of these corneal tissues could alleviate corneal irregularity and enhance riboflavin penetration [29, 30].

The Bowman layer and partial stroma were ablated with the excimer laser in the *CXL-plus-PTK *group, and the regeneration of these structures was limited [31, 32]. The MCT yield change in the *CXL-plus-PTK* group was less effective than in the *Accelerated CXL*group. A previous study found that the cornea gradually thickens up to 3 years postoperatively [33]. Longer term follow-up and larger sample sizes are needed for more definitive evaluation of the long-term efficacies of these modalities.

Contrastingly, the *Iontophoresis CXL* group yielded similar efficacy in the change of keratometry but performed better on the improvement of the **C** value of the ABCD Grading System. The Global Consensus on Keratoconus and Ectatic Diseases (2015) states that no consistent or clear definition of keratoconus progression is available, and acknowledges the lack of specific quantitative data. The ABCD Grading System is increasingly recognized as a more appropriate evaluation system for keratoconus and ectactic corneal disease as the change of the keratometry at a ‘single’ point on the anterior surface, as measured by K_max_, cannot represent the morphological changes of the entire cornea [13, 34, 35]. Presently, clinical studies of the efficacies of different crosslinking procedures according to the more accurate ABCD Grading System are lacking. Our findings suggested that the *Iontophoresis CXL* procedure performed better in reduction of the C value compared with the *Accelerated CXL* group, while the *CXL-plus-PTK* group had decreased performance. Therefore, *Iontophoresis CXL*for 10 min could be the most effective treatment among the five different CXL procedures at 1 year postoperatively. Iontophoresis can effectively deliver appropriate amounts of riboflavin to the stroma through the intact epithelium [36]. Although *Iontophoresis CXL* for 10 min group did not improve keratometry of the anterior surface as much as the *CXL-plus-PTK* group when compared to the reference group, *Iontophoresis CXL* more effectively protected cornea thickness.

Age is an important influence on the effectiveness of CXL [10, 11]. To compensate for the effect of age on CXL efficacy, patients were divided into two age groups (age < 18 years and age ≥ 18 years). After GEE correction, the MCT reduction in pediatric patients was more significant than in adults at 1 year postoperatively following CXL. A prior study reported that pediatric central and paracentral corneal thicknesses increase slowly but did not elaborate on the reason for this phenomenon [37]. However, MCT changes did not significantly differ between adult and pediatric patients at 2 and 4 years after CXL [38, 39]. A longer term follow-up is therefore needed to validate this finding.

Moreover, we found that pediatric patients who underwent mechanical epi-off CXL procedure with a higher UV-A energy (7.2 J/cm^2^) were more likely to develop corneal opacity. Another study that used the same procedure to treat pediatric keratoconus patients did not report complications through 2 years postoperatively [40]. Corneal haze following CXL has been reported in previous studies [41], but the reasons remain unclear at present. Potential reasons for this phenomenon are as follows: (1) more severe corneal ectasis caused by the fibroblast proliferation, which is more common in pediatric patients than in adults due to a more active proliferation response than adults [42, 43]; (2) the haze could be related to slow spontaneous crosslinking reactions triggered by residual riboflavin in the corneal stroma and UV-A rays in natural light [44] as two patients had a history of sunlight exposure early in the postoperative period; or (3) endothelial toxicity caused by reduced corneal thickness [45]. To improve the efficacy of CXL and ensure treatment safety, the *High-Fluence Accelerated CXL* procedure should be applied to pediatric patients with additional caution.

The study has several limitations that should be considered in its interpretation: the lack of a group treated by S-CXL, the accuracy of pre-existing data, and the inherent biases introduced by retrospective analysis. Further, the cornea biomechanics are not completely stable at 1 year following CXL. Hence, these findings should be further confirmed by prospective trials with a longer follow-up period, larger sample size, and better variable selection.

## Conclusion

The *CXL-plus-PTK* procedure was more effective than the *Accelerated CXL* procedure to stop or reverse keratoconus progression based on K_max_. The *Iontophoresis CXL* procedure performed better on **C** value based on the ABCD Grading System. Further, additional improvements to the protocol, such as improved oxygen diffusion with CXL or development of individualized procedures based on corneal topography, could further improve care for patients with keratoconus.

## Supplementary Information

**Additional file 1.**

**Additional file 2.**

**Additional file 3.**

## Data Availability

All data generated or analysed during this study are included in the supplementary files.

## Abbreviations

CXL: Corneal collagen crosslinking
GEE: Generalized estimating equation
PTK: Phototherapeutic keratectomy
Epi-off: Epithelium-off
Epi-on: Epithelium-on
UV: Ultraviolet
UDVA: Uncorrected distance visual acuity
LogMAR: Logarithm of the minimum angle of resolution
CDVA: Corrected distance visual acuity
SE: Spherical equivalence
K_mean_: Mean keratometry
K_max_: Maximum keratometry
MCT: Minimum corneal thickness
ARC: Anterior radius of curvature
A: Staging index for ARC
PRC: Posterior radius of curvature
B: Staging index for PRC,
C: Staging index for MCT
ECD: Endothelial cell density
