# Supplementary material for: Independent-effect comparison of five crosslinking procedures for Progressive Keratoconus based on Keratometry and the ABCD Grading System using Generalized Estimating Equations (GEE)
Source: BMC Ophthalmol. 2023 Jan 10;23:16. doi: 10.1186/s12886-022-02744-w (PMC9830808; doi:10.1186/s12886-022-02744-w)
Supplement: Supplementary file 1 — Additional file 1. [file 12886_2022_2744_MOESM1_ESM.docx]

**Supplemental Digital Content 1-Changes of Clinical Parameters between Baseline and**

**1-year Follow-Up in Five Groups**

|  | **Treatment Procedures** | | | | | | |
| --- | --- | --- | --- | --- | --- | --- | --- |
|  | **Epi-on** | |  | **Epi-off** | | | |
| **Parameter** | **Accelerated Transepithelial CXL** | **Iontophoresis CXL** |  | **CXL-plus-PTK** | **High-Fluence Accelerated CXL** | **Accelerated CXL** |  |
| △UDVA (LogMAR) | -0.02 ± 0.28 | -0.04 ± 0.23 |  | -0.08 ± 0.24^*^ | -0.14 ± 0.23^**^ | -0.18 ± 0.25^**^ |  |
| △CDVA (LogMAR) | -0.03 ± 0.15 | -0.07 ± 0.22^*^ |  | -0.08 ± 0.14^**^ | -0.06 ± 0.13^*^ | -0.07 ± 0.16^*^ |  |
| △SE (D) | 1.01 ± 2.42^*^ | 0.86 ± 3.62^*^ |  | 0.40 ± 2.54 | -0.17 ± 2.27 | 0.13 ± 2.04 |  |
| △K_mean_ (D) | 0.35 ± 0.95^*^ | -0.20 ± 1.55 |  | -0.55 ± 1.04^**^ | -0.10 ± 0.72 | -0.47 ± 0.89^*^ |  |
| △K_max_ (D) | 0.18 ± 2.22 | -1.10 ± 2.97^**^ |  | -2.18 ± 1.70^**^ | -0.67 ± 2.01^*^ | -1.19 ± 1.83^**^ |  |
| ‘Progression’ Rate (n) | 21.43% (9) | 13.33% (10) |  | 0% (0) | 9.76% (4) | 8.82% (3) |  |
| △MCT (μm) | -1 ± 12 | 1 ± 15 |  | -16 ± 13^**^ | -6 ± 16^*^ | -6 ± 26 |  |
| △A | 0.05 ± 0.53 | -0.21 ± 0.92^*^ |  | -0.20 ± 0.95 | -0.21 ± 0.57^*^ | -0.21 ± 0.84 |  |
| △B | 0.03 ± 0.46 | -0.23 ± 0.82^*^ |  | 0.48 ± 0.75^**^ | 0.15 ± 0.58^*^ | 0.05 ± 0.81 |  |
| △C | 0.01 ± 0.24 | -0.07 ± 0.41 |  | 0.32 ± 0.31^**^ | 0.13 ± 0.39^*^ | 0.10 ± 0.36 |  |
| *CXL= corneal crosslinking; PTK= phototherapeutic keratectomy; UDVA= uncorrected distance visual acuity; LogMAR= logarithm of the minimum angle of resolution; CDVA= corrected distance visual acuity; SE= spherical equivalence; D= diopters; K_mean_ = mean keratometry; K_max_= maximum keratometry; MCT= minimum corneal thickness; ARC = anterior radius of curvature, A = staging index for ARC, PRC = posterior radius of curvature, B = staging index for PRC, C = staging index for MCT*  ** indicates* ***P*** *< .05 statistically significant difference compared to baseline; ** indicates* ***P*** *< .001 statistically significant difference compared to baseline.* | | | | | | | |

**Supplemental Digital Content 2- Secondary Outcomes: GEEs Analysis for Changes of UDVA, CDVA, and SE after**

**Five CXL Procedures at 12 Months Postoperatively**

| **Parameter** | **ΔUDVA (LogMAR)** | | |  | **ΔUDVA (LogMAR)** | | |  | **△SE (D)** | | |
| --- | --- | --- | --- | --- | --- | --- | --- | --- | --- | --- | --- |
|  | **β-coefficient^a^** | **95% CI** | ***P*-value^b^** |  | **β-coefficient^a^** | **95% CI** | **P-value^b^** |  | **β-coefficient^a^** | **95% CI** | ***P*-value^b^** |
| Accelerated Transepithelial CXL | 0.052 | -0.056 to 0.160 | 0.347 |  | 0.036 | -0.051 to 0.123 | 0.412 |  | 0.244 | -1.023 to 1.512 | 0.706 |
| Iontophoresis CXL | 0.074 | -0.025 to 0.172 | 0.142 |  | -0.005 | -0.076 to 0.067 | 0.901 |  | 0.129 | -0.804 to 1.063 | 0.786 |
| CXL-plus-PTK | 0.047 | -0.040 to 0.154 | 0.251 |  | -0.011 | -0.069 to 0.046 | 0.701 |  | -0.276 | -1.151 to 0.600 | 0.537 |
| High-Fluence Accelerated CXL | 0.019 | -0.077 to 0.115 | 0.693 |  | -0.002 | -0.058 to 0.054 | 0.950 |  | -0.192 | -1.220 to 0.836 | 0.714 |
| *GEEs= generalized estimating equations; Bold entries are statistically significant (P ＜ 0.05); Each treatment procedures was compared to the reference group (A-CXL= accelerated corneal cross-linking);*  *Δ = difference between 12 months post-CXL and pre-CXL; 95% CI = 95% confidence interval;*  *CXL= corneal crosslinking; PTK= phototherapeutic keratectomy; UDVA= uncorrected distance visual acuity; LogMAR= logarithm of the minimum angle of resolution; CDVA= corrected distance visual acuity; SE= spherical equivalence; D= diopters;*  *^a^The* ***β-coefficient*** *refers to how a dependent variable will change per unit increase in the predictor variable; ^b^****P-value*** *from generalized estimating equations, corrected for baseline.* | | | | | | | | | | | |
