# Supplementary material for: Independent-effect comparison of five crosslinking procedures for Progressive Keratoconus based on Keratometry and the ABCD Grading System using Generalized Estimating Equations (GEE)
Source: BMC Ophthalmol. 2023 Jan 10;23:16. doi: 10.1186/s12886-022-02744-w (PMC9830808; doi:10.1186/s12886-022-02744-w)
Supplement: Supplementary file 2 — Additional file 2. [file 12886_2022_2744_MOESM2_ESM.docx]

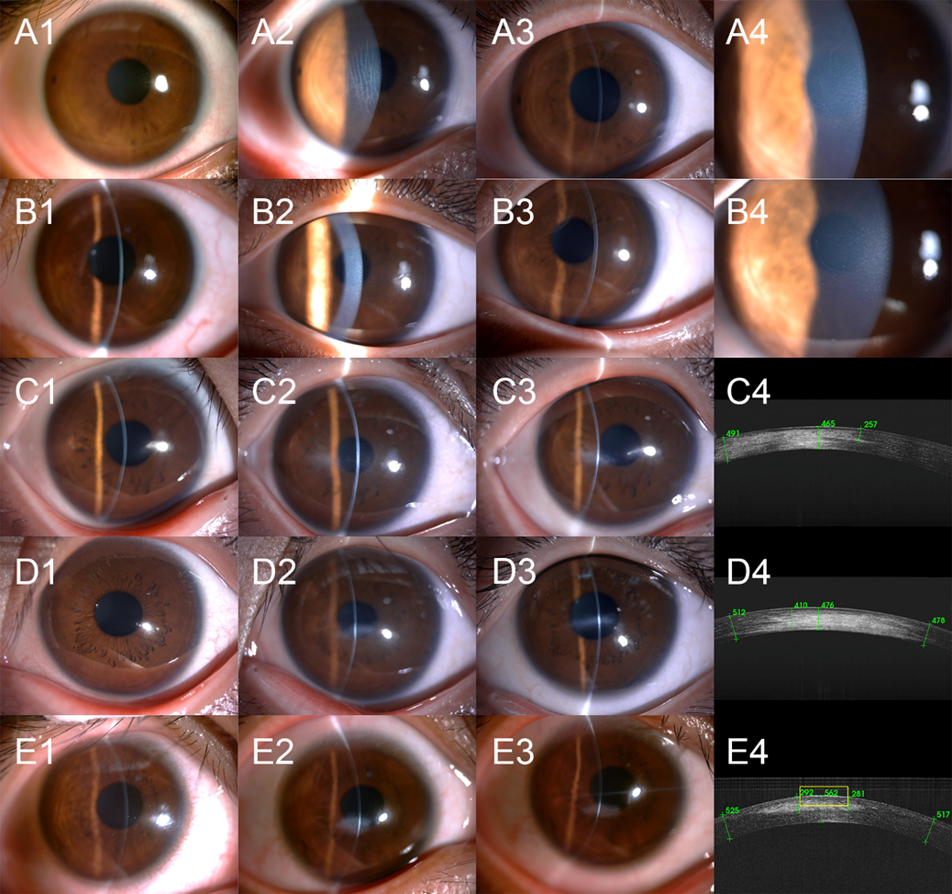


**Supplementary Figure.** Opacity of the corneal stroma in five eyes

**Note:** AB is the patient's right and left eyes, A1 and B1 are preoperative slit lamp micrographs, A2 and B2 are the first time the patient was seen on follow-up. The corneal clouding was seen as vertical streak-like clouding in A2 and diffuse dot-like clouding in B2 (11 days after surgery in A2 and 47 days after surgery in B2). (A2 is 11 days postoperative, B2 is 47 days postoperative, where B2 had corneal clouding on 14 days postoperative, but because the patient was 10 years old, the picture was not taken well on that day. However, because the patient was 10 years old, the picture was not taken on that day, so it was replaced with a picture taken 47 days after surgery, at which time the clouding was not reduced. CD shows the patient's second right eye and left eye. C1 and D1 are preoperative photographs, while C2 and D2 are photographs of the first corneal clouding found at follow-up. (47 days postoperative and 40 days postoperative, respectively), C3, D3, and C4, D4 are end-of-study photographs and optical coherence tomography, respectively. C, D3, and C4, D4 are end-of-study photographic and optical coherence tomography findings (13 months postoperatively), respectively, which show a slight reduction of the clouding after aggressive intervention, but still a mid- to deep stromal clouding. E is the right eye of patient three, who had cross-linking procedures performed 5 days apart in both eyes until the last follow-up, with only the right eye showing stromal clouding and the left eye showing stromal clouding. E1 is a preoperative photograph and E2 is a postoperative follow-up photograph of the first corneal clouding. E1 is a preoperative photograph and E2 is a postoperative follow-up photograph of the first corneal clouding, which shows a lamellar superficial to medium stromal clouding (126 days postoperatively). (11 months postoperatively), it can be seen that the superficial mesial stromal clouding faded significantly after active intervention.
