# Supplementary material for: Independent-effect comparison of five crosslinking procedures for Progressive Keratoconus based on Keratometry and the ABCD Grading System using Generalized Estimating Equations (GEE)
Source: BMC Ophthalmol. 2023 Jan 10;23:16. doi: 10.1186/s12886-022-02744-w (PMC9830808; doi:10.1186/s12886-022-02744-w)
Supplement: Supplementary file 3 — Additional file 3. [file 12886_2022_2744_MOESM3_ESM.docx]

Raw Data of ‘Independent-effect Comparison of Five Crosslinking Procedures for Progressive Keratoconus Based on

Keratometry and the ABCD Grading System Using Generalized Estimating Equations (GEE)’

(**sex**: 1=male, 2=female; **procedure**: 1= Accelerated Transepithelial CXL, 2=Iontophoresis CXL, 3=CXL-plus-PTK, 4=High-Fluence Accelerated CXL, 5=Accelerated CXL;

**eye**: 1=right eye, 2=left eye)

| No. | sex | age | procedure | eye | **UDVA (LogMAR)** | | | **CDVA (LogMAR)** | | | **SE (D)** | | | **Kmean (D)** | | | **Kmax (D)** | | | **MCT (μm)** | | | **A** | | | **B** | | | **C** | | |
| --- | --- | --- | --- | --- | --- | --- | --- | --- | --- | --- | --- | --- | --- | --- | --- | --- | --- | --- | --- | --- | --- | --- | --- | --- | --- | --- | --- | --- | --- | --- | --- |
|  |  |  |  |  | **pre** | **post** | △ | **pre** | **post** | △ | **pre** | **post** | △ | **pre** | **post** | △ | **pre** | **post** | △ | **pre** | **post** | **△** | **pre** | **post** | △ | **pre** | **post** | △ | **pre** | **post** | △ |
| 1 | 2 | 16 | 1 | 1 | **0.9** | 1 | 0.1 | **0.3** | 0.5 | 0.2 | **-6** | -8.75 | -2.75 | **58.1** | 58.4 | 0.3 | **78.7** | 76.1 | -2.6 | **450** | 454 | 4 | **8.65** | 8.87 | 0.22 | **11.04** | 10.38 | -0.66 | **1.99** | 1.94 | -0.05 |
| 15 | 1 | 15 | 1 | 1 | **0.6** | 0.3 | -0.30 | **0.6** | 0.8 | 0.20 | **-5.25** | -4.375 | 0.875 | **45.3** | 45.1 | -0.2 | **50.3** | 51.3 | 1.0 | **555** | 530 | -25 | **0.93** | 1.51 | 0.58 | **2.46** | 2.49 | 0.03 | **0.07** | 0.43 | 0.36 |
| 2 | 1 | 27 | 1 | 2 | **0.7** | 0.7 | 0.00 | **0.2** | 0.2 | 0.00 | **-6.125** | -6.375 | -0.250 | **48.9** | 49.1 | 0.2 | **59.4** | 59 | -0.4 | **456** | 478 | 22 | **3.7** | 3.47 | -0.23 | **6.08** | 5.54 | -0.54 | **1.85** | 1.45 | -0.4 |
| 100 | 1 | 29 | 1 | 1 | **0.25** | 0.5 | 0.25 | **0.6** | 0.8 | 0.20 | **-6.50** | -5.875 | 0.625 | **50.4** | 50.5 | 0.1 | **58.2** | 57.8 | -0.4 | **439** | 444 | 5 | **2.82** | 2.77 | -0.05 | **4.7** | 4.56 | -0.14 | **2.21** | 2.13 | -0.08 |
| 100 | 1 | 29 | 1 | 2 | **0.3** | 0.4 | 0.10 | **0.4** | 0.8 | 0.40 | **-7.00** | -6.50 | 0.500 | **51.9** | 52.1 | 0.2 | **61.6** | 61.4 | -0.2 | **423** | 428 | 5 | **3.49** | 3.27 | -0.22 | **5.79** | 5.92 | 0.13 | **2.53** | 2.44 | -0.09 |
| 17 | 1 | 15 | 1 | 1 | **0.12** | 0.2 | 0.08 | **0.5** | 0.5 | 0.00 | **-8.25** | -4.875 | 3.375 | **52.3** | 51.8 | -0.5 | **59.1** | 59 | -0.1 | **436** | 439 | 3 | **4.24** | 3.81 | -0.43 | **5.57** | 4.76 | -0.81 | **2.27** | 2.05 | -0.22 |
| 44 | 1 | 18 | 1 | 1 | **0.40** | 0.30 | -0.10 | **0.40** | 0.10 | -0.30 | **-6.00** | -6.375 | -0.375 | **47.1** | 46.4 | -0.7 | **58.5** | 56.4 | -2.1 | **448** | 450 | 2 | **2.6** | 2.45 | -0.15 | **4.46** | 4.88 | 0.42 | **2.03** | 2 | -0.03 |
| 8 | 1 | 13 | 1 | 2 | **0.00** | -0.10 | -0.10 | **-0.10** | -0.10 | 0.00 | **0** | -0.125 | -0.125 | **45.7** | 46 | 0.3 | **56.9** | 58.1 | 1.2 | **440** | 443 | 3 | **2.3** | 2.4 | 0.1 | **3.05** | 3.27 | 0.22 | **2.19** | 2.14 | -0.05 |
| 45 | 1 | 18 | 1 | 2 | **0.90** | 1.10 | 0.20 | **0.10** | 0.20 | 0.10 | **-9.00** | -8.50 | 0.500 | **48.8** | 48.6 | -0.2 | **59.6** | 57.7 | -1.9 | **429** | 425 | -4 | **4.78** | 4.05 | -0.73 | **7.24** | 7.37 | 0.13 | **2.42** | 2.51 | 0.09 |
| 101 | 2 | 29 | 1 | 1 | **0.80** | 1.10 | 0.30 | **0.20** | 0.10 | -0.10 | **-6.75** | -7.50 | -0.750 | **49.4** | 50.1 | 0.7 | **55.7** | 54.5 | -1.2 | **457** | 473 | 16 | **2.93** | 2.85 | -0.08 | **4.55** | 4.6 | 0.05 | **1.82** | 1.41 | -0.41 |
| 82 | 1 | 23 | 1 | 1 | **1.10** | 0.80 | -0.30 | **0.20** | 0.30 | 0.10 | **-18.00** | -18.50 | -0.500 | **54.6** | 55.2 | 0.6 | **61.7** | 62 | 0.3 | **440** | 454 | 14 | **4.91** | 5 | 0.09 | **7.32** | 7.29 | -0.03 | **2.2** | 1.89 | -0.31 |
| 107 | 1 | 37 | 1 | 2 | **1.30** | 1.00 | -0.30 | **0.10** | 0.10 | 0.00 | **-17.375** | -17.50 | -0.125 | **51.5** | 50.9 | -0.6 | **54.5** | 53.2 | -1.3 | **453** | 436 | -17 | **2.97** | 2.69 | -0.28 | **4.41** | 4.06 | -0.35 | **1.93** | 2.27 | 0.34 |
| 58 | 1 | 20 | 1 | 2 | **0.60** | 0.40 | -0.20 | **0.10** | 0.00 | -0.10 | **-7.50** | -8.50 | -1.000 | **43.5** | 43.8 | 0.3 | **50.8** | 52.4 | 1.6 | **486** | 471 | -15 | **0.6** | 0.74 | 0.14 | **2.15** | 2.18 | 0.03 | **1.09** | 1.48 | 0.39 |
| 59 | 2 | 20 | 1 | 1 | **0.90** | 1.10 | 0.20 | **0.30** | 0.20 | -0.10 | **-7.125** | -7.375 | -0.250 | **54.7** | 53.7 | -1.0 | **65.8** | 63.4 | -2.4 | **428** | 414 | -14 | **5.7** | 5.83 | 0.13 | **4.12** | 4.1 | -0.02 | **5.57** | 5.99 | 0.42 |
| 74 | 1 | 22 | 1 | 1 | **0.60** | 0.60 | 0.00 | **0.30** | 0.20 | -0.10 | **-4.75** | -4.75 | 0.000 | **44.1** | 44.4 | 0.3 | **45.7** | 46.1 | 0.4 | **485** | 488 | 3 | **0** | 0.17 | 0.17 | **0** | 0 | 0 | **1.11** | 1.05 | -0.06 |
| 51 | 1 | 19 | 1 | 1 | **0.70** | 0.60 | -0.10 | **0.00** | 0.10 | 0.10 | **-5.75** | -8.50 | -2.750 | **46.1** | 46.3 | 0.2 | **52.4** | 52.5 | 0.1 | **444** | 451 | 7 | **2.19** | 2.23 | 0.04 | **2.8** | 2.75 | -0.05 | **2.12** | 1.96 | -0.16 |
| 51 | 1 | 19 | 1 | 2 | **0.90** | 0.80 | -0.10 | **0.20** | 0.20 | 0.00 | **-15.125** | -13.50 | 1.625 | **52.6** | 52.8 | 0.2 | **60.1** | 61.1 | 1.0 | **399** | 407 | 8 | **4.23** | 4.4 | 0.17 | **6.1** | 6.03 | -0.07 | **3.01** | 2.85 | -0.16 |
| 75 | 1 | 22 | 1 | 2 | **0.12** | 0.5 | 0.38 | **0.5** | 0.8 | 0.30 | **-4.25** | -2.75 | 1.500 | **44.5** | 46.9 | 2.4 | **57.3** | 60.2 | 2.9 | **408** | 413 | 5 | **2.21** | 2.6 | 0.39 | **2.26** | 2.53 | 0.27 | **2.84** | 2.74 | -0.1 |
| 35 | 1 | 17 | 1 | 2 | **0.4** | 0 | -0.40 | **0.1** | 0 | -0.10 | **-1.875** | -0.50 | 1.375 | **44.1** | 44.1 | 0.0 | **51.6** | 51.7 | 0.1 | **439** | 450 | 11 | **1.5** | 0.97 | -0.53 | **2.13** | 2.08 | -0.05 | **2.22** | 1.99 | -0.23 |
| 103 | 1 | 30 | 1 | 1 | **0.50** | 0.40 | -0.10 | **0.50** | 0.40 | -0.10 | **-0.50** | -0.625 | -0.125 | **53.8** | 54.8 | 1.0 | **73.5** | 74.3 | 0.8 | **448** | 449 | 1 | **2.65** | 2.75 | 0.1 | **4.78** | 4.59 | -0.19 | **2.05** | 2.01 | -0.04 |
| 103 | 1 | 30 | 1 | 2 | **0.70** | 0.80 | 0.10 | **0.70** | 0.70 | 0.00 | **3.625** | 3.75 | 0.125 | **44.4** | 44.5 | 0.1 | **48.5** | 49 | 0.5 | **494** | 491 | -3 | **0.11** | 0.35 | 0.24 | **0.12** | 0.37 | 0.25 | **0.93** | 0.98 | 0.05 |
| 89 | 1 | 24 | 1 | 1 | **1.00** | 0.80 | -0.20 | **0.30** | 0.20 | -0.10 | **-11.25** | -8.125 | 3.125 | **49** | 48.7 | -0.3 | **57.2** | 56.7 | -0.5 | **496** | 503 | 7 | **2.87** | 2.74 | -0.13 | **4.74** | 4.53 | -0.21 | **0.9** | 0.81 | -0.09 |
| 154 | 2 | 35 | 1 | 1 | **0.80** | 0.40 | -0.40 | **0.50** | 0.40 | -0.10 | **-7.125** | -6.50 | 0.625 | **51.8** | 51.9 | 0.1 | **63.4** | 63 | -0.4 | **415** | 414 | -1 | **3.01** | 4.16 | 1.15 | **6.99** | 6.74 | -0.25 | **2.69** | 2.72 | 0.03 |
| 114 | 1 | 14 | 1 | 1 | **0.60** | 0.80 | 0.20 | **0.10** | 0.00 | -0.10 | **-8.25** | -5.50 | 2.750 | **44.8** | 44.8 | 0.0 | **53.4** | 51.8 | -1.6 | **426** | 420 | -6 | **1.69** | 1.29 | -0.4 | **2.63** | 2.6 | -0.03 | **2.47** | 2.6 | 0.13 |
| 114 | 1 | 14 | 1 | 2 | **0.80** | 0.80 | 0.00 | **0.20** | 0.00 | -0.20 | **-8.50** | -5.00 | 3.500 | **44.6** | 44.7 | 0.1 | **53.7** | 52.7 | -1.0 | **438** | 432 | -6 | **1.51** | 1.21 | -0.3 | **2.36** | 2.44 | 0.08 | **2.23** | 2.35 | 0.12 |
| 111 | 1 | 10 | 1 | 1 | **0.30** | 0.40 | 0.10 | **0.30** | 0.30 | 0.00 | **-5.375** | -0.50 | 4.875 | **55.2** | 58.4 | 3.2 | **66** | 70 | 4.0 | **443** | 403 | -40 | **5.83** | 6.87 | 1.04 | **7.86** | 9.1 | 1.24 | **2.14** | 2.93 | 0.79 |
| 111 | 1 | 10 | 1 | 2 | **0.90** | 0.40 | -0.50 | **0.40** | 0.30 | -0.10 | **-10.50** | -1.50 | 9.000 | **56.1** | 58.7 | 2.6 | **69** | 70.3 | 1.3 | **438** | 415 | -23 | **7.23** | 7.12 | -0.11 | **9.15** | 9.11 | -0.04 | **2.23** | 2.69 | 0.46 |
| 115 | 2 | 14 | 1 | 1 | **0.70** | 0.90 | 0.20 | **0.40** | 0.30 | -0.10 | **-14.75** | -14.75 | 0.000 | **52.7** | 51.2 | -1.5 | **63.4** | 55.8 | -7.6 | **391** | 389 | -2 | **4.44** | 2.89 | -1.55 | **6.74** | 6.19 | -0.55 | **3.09** | 3.11 | 0.02 |
| 115 | 2 | 14 | 1 | 2 | **1.00** | 0.90 | -0.10 | **0.40** | 0.30 | -0.10 | **-13.375** | -14.50 | -1.125 | **48.7** | 51.2 | 2.5 | **54.1** | 58.6 | 4.5 | **374** | 379 | 5 | **2.35** | 3.61 | 1.26 | **5.29** | 6.66 | 1.37 | **3.26** | 3.21 | -0.05 |
| 149 | 2 | 31 | 1 | 1 | **1.10** | 1.70 | 0.60 | **0.30** | 0.30 | 0.00 | **-19.000** | -22.75 | -3.750 | **49.4** | 49.4 | 0.0 | **54.1** | 53.5 | -0.6 | **396** | 401 | 5 | **2.53** | 2.49 | -0.04 | **2.92** | 3 | 0.08 | **3.13** | 2.98 | -0.15 |
| 128 | 1 | 18 | 1 | 1 | **0.90** | 0.80 | -0.10 | **0.40** | 0.30 | -0.10 | **-8.000** | -1.250 | 6.750 | **54.6** | 56.3 | 1.7 | **72.1** | 77 | 4.9 | **423** | 417 | -6 | **6.59** | 7.3 | 0.71 | **8.08** | 8.66 | 0.58 | **2.54** | 2.65 | 0.11 |
| 128 | 1 | 18 | 1 | 2 | **0.50** | 0.30 | -0.20 | **0.30** | 0.10 | -0.20 | **-1.500** | -0.500 | 1.000 | **51.8** | 52.7 | 0.9 | **69.1** | 74.7 | 5.6 | **437** | 428 | -9 | **4.56** | 5.81 | 1.25 | **6.97** | 8.06 | 1.09 | **2.26** | 2.43 | 0.17 |
| 133 | 1 | 19 | 1 | 1 | **0.40** | 0.30 | -0.10 | **0.00** | -0.10 | -0.10 | **-5.750** | -3.000 | 2.750 | **45.6** | 45.9 | 0.3 | **57.1** | 56.5 | -0.6 | **450** | 456 | 6 | **2.25** | 2.19 | -0.06 | **3.19** | 3.78 | 0.59 | **2** | 1.83 | -0.17 |
| 133 | 1 | 19 | 1 | 2 | **0.50** | 0.40 | -0.10 | **0.20** | 0.20 | 0.00 | **-7.000** | -4.000 | 3.000 | **48.7** | 48.3 | -0.4 | **60.6** | 60.6 | 0.0 | **427** | 440 | 13 | **2.98** | 2.96 | -0.02 | **6.08** | 6.05 | -0.03 | **2.46** | 2.2 | -0.26 |
| 112 | 1 | 13 | 1 | 2 | **0.30** | 0.50 | 0.20 | **0.30** | 0.20 | -0.10 | **0.000** | -1.250 | -1.250 | **57.2** | 56.8 | -0.4 | **77.5** | 75.3 | -2.2 | **391** | 390 | -1 | **8.39** | 7.69 | -0.7 | **9.17** | 8.73 | -0.44 | **3.08** | 3.09 | 0.01 |
| 152 | 2 | 34 | 1 | 1 | **0.90** | 0.70 | -0.20 | **0.10** | 0.30 | 0.20 | **-7.125** | -6.125 | 1.000 | **47.9** | 48.6 | 0.7 | **54.7** | 55.8 | 1.1 | **389** | 398 | 9 | **2.5** | 2.62 | 0.12 | **3.61** | 3.9 | 0.29 | **3.1** | 3.02 | -0.08 |
| 152 | 2 | 34 | 1 | 2 | **0.80** | 0.70 | -0.10 | **0.10** | 0.00 | -0.10 | **-5.500** | -4.375 | 1.125 | **44.5** | 44.6 | 0.1 | **47.4** | 47.7 | 0.3 | **427** | 429 | 2 | **0.29** | 0.39 | 0.1 | **0.87** | 0.91 | 0.04 | **2.46** | 2.42 | -0.04 |
| 129 | 1 | 19 | 2 | 2 | **0.9** | 0.9 | 0.00 | **0.4** | 0.1 | -0.30 | **-8.00** | -5.75 | 2.250 | **52.8** | 52.7 | -0.1 | **74.7** | 71.1 | -3.6 | **422** | 412 | -10 | **5.49** | 6.28 | 0.79 | **6.79** | 6.07 | -0.72 | **2.56** | 2.47 | -0.09 |
| 76 | 1 | 22 | 2 | 2 | **0.4** | 0.3 | -0.10 | **0.2** | 0 | -0.20 | **-7.50** | -7.875 | -0.375 | **47.3** | 47.5 | 0.2 | **53.7** | 54.8 | 1.1 | **468** | 454 | -14 | **2.35** | 2.37 | 0 | **3.08** | 2.95 | -0 | **1.55** | 1.89 | 0 |
| 12 | 1 | 15 | 2 | 1 | **1.7** | 1 | -0.70 | **0.4** | 0.3 | -0.10 | **-3.375** | -6.625 | -3.250 | **59.1** | 58.1 | -1.0 | **72.6** | 72.7 | 0.1 | **384** | 366 | -18 | **7.97** | 7 | -1 | **11.11** | 11.56 | 0 | **3.16** | 3.34 | 0 |
| 43 | 1 | 18 | 2 | 1 | **1** | 1 | 0.00 | **0.3** | 0.3 | 0.00 | **-4.125** | -9.75 | -5.625 | **52.1** | 52.5 | 0.4 | **62.1** | 62.1 | 0.0 | **457** | 469 | 12 | **4.51** | 4.38 | -0 | **6.75** | 6.19 | -1 | **1.82** | 1.53 | -0 |
| 119 | 2 | 15 | 2 | 1 | **0.6** | 0.3 | -0.30 | **0.3** | 0.2 | -0.10 | **-4.625** | -6.25 | -1.625 | **48.9** | 48.3 | -0.6 | **61.8** | 60.7 | -1.1 | **388** | 408 | 20 | **2.92** | 2.64 | -0 | **5.32** | 4.39 | -1 | **2.74** | 2.97 | 0 |
| 16 | 1 | 15 | 2 | 2 | **0.8** | 1 | 0.20 | **0.8** | 0.3 | -0.50 | **-11.00** | -7.50 | 3.500 | **47.7** | 51.5 | 3.8 | **54.7** | 62.8 | **8.1** | **460** | 461 | 1 | **2.82** | 4.13 | 1.31 | **5.05** | 5.5 | 0.45 | **1.74** | 1.72 | -0.02 |
| 7 | 1 | 13 | 2 | 1 | **0.7** | 0.9 | 0.20 | **0.1** | 0.1 | 0.00 | **-7.00** | -7.50 | -0.500 | **46.6** | 46.5 | -0.1 | **51.1** | 51.1 | 0.0 | **462** | 462 | 0 | **2** | 1.84 | -0.16 | **2.47** | 2.47 | 0 | **1.7** | 1.65 | -0.05 |
| 7 | 1 | 13 | 2 | 2 | **0.4** | 0.4 | 0.00 | **0.2** | 0.2 | 0.00 | **-6.75** | -7.25 | -0.500 | **50.5** | 50.7 | 0.2 | **59.7** | 58.8 | -0.9 | **435** | 439 | 4 | **3.2** | 2.96 | -0.24 | **5.18** | 5.01 | -0.17 | **2.29** | 2.19 | -0.1 |
| 138 | 2 | 22 | 2 | 1 | **0.4** | 0.4 | 0.00 | **0.2** | 0.2 | 0.00 | **-4.75** | -4.50 | 0.250 | **50** | 50.6 | 0.5 | **61.9** | 62.5 | 0.6 | **462** | 457 | -5 | **3.94** | 4.4 | 0.46 | **7.38** | 7.7 | 0.32 | **1.69** | 1.83 | 0.14 |
| 138 | 2 | 22 | 2 | 2 | **0.4** | 0.3 | -0.10 | **0** | 0 | 0.00 | **-2.00** | -2.00 | 0.000 | **44.4** | 44.5 | 0.1 | **46** | 46.3 | 0.3 | **496** | 496 | 0 | **0.68** | 0.74 | 0.06 | **2.69** | 2.64 | -0.05 | **0.91** | 0.91 | 0 |
| 90 | 1 | 24 | 2 | 1 | **0.7** | 0.4 | -0.30 | **0.3** | 0 | -0.30 | **-4.50** | -4.875 | -0.375 | **47.7** | 45.3 | -2.4 | **50.4** | 49.1 | -1.3 | **402** | 416 | 14 | **2.19** | 0.45 | -1.74 | **5.47** | 6.17 | 0.7 | **2.95** | 2.68 | -0.27 |
| 25 | 2 | 16 | 2 | 1 | **0.4** | 0.1 | -0.30 | **0.1** | 0 | -0.10 | **-0.875** | -1.00 | -0.125 | **46.4** | 46.9 | 0.5 | **51** | 51.5 | 0.5 | **476** | 475 | -1 | **1.69** | 1.92 | 0.23 | **2.08** | 2.26 | 0.18 | **1.35** | 1.36 | 0.01 |
| 25 | 2 | 16 | 2 | 2 | **0.4** | 0.4 | 0.00 | **0.2** | 0.3 | 0.10 | **-3.50** | -4.25 | -0.750 | **49.2** | 49.9 | 0.7 | **58.2** | 58.9 | 0.7 | **423** | 420 | -3 | **3.6** | 4.16 | 0.56 | **4.32** | 4.65 | 0.33 | **2.54** | 2.6 | 0.06 |
| 77 | 1 | 22 | 2 | 1 | **1.1** | 1.4 | 0.30 | **0.3** | 0.3 | 0.00 | **-9.25** | -9.50 | -0.250 | **52.4** | 50.7 | -1.8 | **61.4** | 57.4 | -4.0 | **397** | 386 | -11 | **4.39** | 3.04 | -1.35 | **7.49** | 7.59 | 0.1 | **3.02** | 3.14 | 0.12 |
| 78 | 2 | 22 | 2 | 1 | **0.3** | 0.4 | 0.10 | **0.1** | 0 | -0.10 | **-7.00** | -5.75 | 1.250 | **46.5** | 47.9 | 1.4 | **51.7** | 52.8 | **1.1** | **433** | 424 | -9 | **2.21** | 2.43 | 0.22 | **2.79** | 2.99 | 0.2 | **2.33** | 2.51 | 0.18 |
| 26 | 1 | 16 | 2 | 2 | **0.7** | 1 | 0.30 | **0** | 0 | 0.00 | **-6.25** | -6.875 | -0.625 | **46.2** | 45.3 | -1.0 | **51.3** | 49 | -2.3 | **517** | 510 | -7 | **0.14** | 0.37 | 0.23 | **0.42** | 0 | -0.42 | **0.6** | 0.7 | 0.1 |
| 27 | 1 | 16 | 2 | 2 | **0.4** | 0 | -0.40 | **0.1** | 0 | -0.10 | **-1.875** | -1.625 | 0.250 | **45.2** | 44.9 | -0.3 | **51.6** | 51.2 | -0.4 | **455** | 456 | 1 | **1.35** | 1.17 | -0.18 | **2.39** | 2.44 | 0.05 | **1.86** | 1.84 | -0.02 |
| 5 | 1 | 11 | 2 | 1 | **0.4** | 0.4 | 0.00 | **0** | 0 | 0.00 | **-6.75** | -7.125 | -0.375 | **45.5** | 45.3 | -0.2 | **48.6** | 47.7 | -0.9 | **498** | 493 | -5 | **0.51** | 0.49 | -0.02 | **1.31** | 1.19 | -0.12 | **0.88** | 0.96 | 0.08 |
| 36 | 1 | 17 | 2 | 1 | **0.4** | 0.4 | 0.00 | **0.4** | 0.2 | -0.20 | **-8.25** | -8.75 | -0.500 | **50.2** | 51.3 | 1.1 | **62.6** | 62.8 | 0.2 | **407** | 429 | 22 | **2.84** | 3.72 | 0.88 | **5.92** | 6.01 | 0.09 | **2.85** | 2.41 | -0.44 |
| 37 | 1 | 17 | 2 | 1 | **1.4** | 1.7 | 0.30 | **0.3** | 0.1 | -0.20 | **-14.50** | -13.25 | 1.250 | **53.5** | 52.6 | -0.9 | **61.9** | 57.1 | -4.8 | **398** | 409 | 11 | **3.65** | 2.93 | -0.72 | **6.03** | 4.99 | -1.04 | **3.01** | 2.82 | -0.19 |
| 91 | 1 | 24 | 2 | 1 | **0.7** | 0.6 | -0.10 | **0** | 0 | 0.00 | **-2.25** | -2.50 | -0.250 | **42.9** | 43.2 | 0.3 | **44.5** | 44.6 | 0.1 | **498** | 470 | -28 | **0** | 0 | 0 | **0.01** | 0 | -0.01 | **0.89** | 1.13 | 0.24 |
| 11 | 1 | 14 | 2 | 1 | **1.1** | 0.9 | -0.20 | **0.1** | 0.1 | 0.00 | **-9.00** | -9.125 | -0.125 | **47.8** | 47.6 | -0.2 | **59.5** | 57.9 | -1.6 | **429** | 420 | -9 | **2.5** | 2.4 | -0.1 | **3** | 3.1 | 0.1 | **2.4** | 2.6 | 0.2 |
| 11 | 1 | 14 | 2 | 2 | **0.9** | 0.9 | 0.00 | **0** | 0 | 0.00 | **-7.875** | -7.625 | 0.250 | **45.2** | 45.1 | -0.1 | **50.4** | 49.3 | -1.1 | **440** | 443 | 3 | **0.9** | 0.7 | -0.2 | **2** | 2.1 | 0.1 | **2.2** | 2.1 | -0.1 |
| 60 | 2 | 21 | 2 | 1 | **1** | 1 | 0.00 | **0.4** | 0.4 | 0.00 | **-3.625** | -3.375 | 0.250 | **53.3** | 53.0 | -0.3 | **66.9** | 65.5 | -1.4 | **468** | 467 | -1 | **5.19** | 4.87 | -0.32 | **6.82** | 6.59 | -0.23 | **1.54** | 1.6 | 0.06 |
| 67 | 2 | 22 | 2 | 1 | **1.2** | 1.3 | 0.10 | **1** | 0.8 | -0.20 | **-9.00** | -9.25 | -0.250 | **50.4** | 51.0 | 0.6 | **56.3** | 56.7 | 0.4 | **451** | 438 | -13 | **2.74** | 2.8 | 0.06 | **4.17** | 4.22 | 0.05 | **1.96** | 2.22 | 0.26 |
| 102 | 2 | 30 | 2 | 2 | **0.7** | 1 | 0.30 | **0.4** | 0.3 | -0.10 | **-12.00** | -10.25 | 1.750 | **49.5** | 49.5 | 0.0 | **55.7** | 57 | **1.3** | **409** | 404 | -5 | **2.74** | 2.84 | 0.1 | **4.8** | 4.9 | 0.1 | **2.81** | 2.92 | 0.11 |
| 68 | 1 | 22 | 2 | 1 | **0.6** | 0.4 | -0.20 | **0.3** | 0 | -0.30 | **-4.50** | -4.25 | 0.250 | **45** | 44.7 | -0.3 | **49.1** | 47.8 | -1.3 | **512** | 506 | -6 | **0.57** | 0.51 | -0.06 | **0.64** | 1 | 0.36 | **0.68** | 0.76 | 0.08 |
| 61 | 1 | 21 | 2 | 2 | **1.1** | 1.3 | 0.20 | **0.2** | 0.1 | -0.10 | **-8.00** | -5.25 | 2.750 | **50.3** | 50.6 | 0.3 | **57** | 57.5 | 0.5 | **501** | 496 | -5 | **2.69** | 2.66 | -0.03 | **3.59** | 3.54 | -0.05 | **0.84** | 0.91 | 0.07 |
| 39 | 1 | 18 | 2 | 1 | **1.1** | 1.2 | 0.10 | **0.3** | 0.4 | 0.10 | **-14.375** | -14.50 | -0.125 | **54.8** | 54.8 | -0.0 | **64.6** | 61.8 | -2.8 | **444** | 452 | 8 | **3.52** | 4.19 | 0.67 | **9.2** | 8.76 | -0.44 | **2.12** | 1.91 | -0.21 |
| 69 | 1 | 22 | 2 | 2 | **0.3** | 0.2 | -0.10 | **0.1** | 0 | -0.10 | **-2.50** | 0.50 | 3.000 | **42.9** | 42.5 | -0.4 | **49.6** | 49 | -0.6 | **440** | 436 | -4 | **1.36** | 1.2 | -0.16 | **2.5** | 2.44 | -0.06 | **2.2** | 2.28 | 0.08 |
| 18 | 1 | 16 | 2 | 1 | **0.8** | 1 | 0.20 | **0.3** | 0.4 | 0.10 | **-5.125** | -4.125 | 1.000 | **53.6** | 55.0 | 1.4 | **65** | 67 | **2.0** | **416** | 423 | 7 | **6.45** | 6.79 | 0.34 | **8.75** | 8.33 | -0.42 | **2.68** | 2.53 | -0.15 |
| 18 | 1 | 16 | 2 | 2 | **0.8** | 0.9 | 0.10 | **0.3** | 0.4 | 0.10 | **-5.125** | -5.00 | 0.125 | **51.2** | 51.3 | 0.1 | **58.5** | 56.7 | -1.8 | **433** | 425 | -8 | **3.77** | 3.18 | -0.59 | **6.13** | 6.25 | 0.12 | **2.33** | 2.49 | 0.16 |
| 79 | 1 | 23 | 2 | 2 | **1** | 1 | 0.00 | **0.3** | 0.7 | 0.40 | **-4.00** | -4.00 | 0.000 | **47.4** | 47.8 | 0.4 | **55.9** | 55.1 | -0.8 | **440** | 441 | 1 | **2.15** | 2.01 | -0.14 | **2.95** | 2.91 | -0.04 | **2.19** | 2.17 | -0.02 |
| 46 | 1 | 19 | 2 | 1 | **0.4** | 0.3 | -0.10 | **0** | 0 | 0.00 | **-2.875** | -1.25 | 1.625 | **48.3** | 47.9 | -0.5 | **57.4** | 55.6 | -1.8 | **467** | 483 | 16 | **2.99** | 2.74 | -0.25 | **4.88** | 3.47 | -1.41 | **1.52** | 1.17 | -0.35 |
| 46 | 1 | 19 | 2 | 2 | **1.7** | 1 | -0.70 | **0.3** | 0.4 | 0.10 | **-6.25** | -5.75 | 0.500 | **64** | 64.8 | 0.8 | **76.4** | 74.9 | -1.5 | **409** | 366 | -43 | **10.5** | 10.33 | -0.17 | **11.97** | 12 | 0.03 | **2.82** | 3 | 0.18 |
| 19 | 1 | 16 | 2 | 2 | **1.5** | 1 | -0.50 | **0.9** | 0.1 | -0.80 | **-20.00** | -6.875 | 13.125 | **54.2** | 50.9 | -3.3 | **66.4** | 58.3 | -8.1 | **436** | 387 | -49 | **7.18** | 2.93 | -4.25 | **8.96** | 8.54 | -0.42 | **2.28** | 3 | 0.72 |
| 108 | 2 | 39 | 2 | 2 | **0.6** | 0.4 | -0.20 | **0.4** | 0.2 | -0.20 | **-3.375** | -1.875 | 1.500 | **45.5** | 44.7 | -0.8 | **57.1** | 55.2 | -1.9 | **465** | 457 | -8 | **2.49** | 2.47 | -0.02 | **3.58** | 4.18 | 0.6 | **1.62** | 1.01 | -0.61 |
| 20 | 1 | 16 | 2 | 1 | **0.8** | 0.6 | -0.20 | **0** | 0.1 | 0.10 | **-6.875** | -7.25 | -0.375 | **43.5** | 43.6 | 0.1 | **44.5** | 44.5 | 0.0 | **474** | 454 | -20 | **0** | 0 | 0 | **0** | 0 | 0 | **1.4** | 1.89 | 0.49 |
| 62 | 1 | 21 | 2 | 2 | **1.5** | 1 | -0.50 | **0.7** | 0.3 | -0.40 | **-11.00** | -9.50 | 1.500 | **50.2** | 51.3 | 1.1 | **60.9** | 64.7 | **3.8** | **460** | 464 | 4 | **5.54** | 4.48 | -1.06 | **7.58** | 7.12 | -0.46 | **2.36** | 1.64 | -0.72 |
| 89 | 1 | 24 | 2 | 2 | **1** | 1 | 0.00 | **1** | 0.8 | -0.20 | **-7.50** | -8.125 | -0.625 | **66.8** | 69.1 | 2.3 | **83.2** | 85.1 | **1.9** | **397** | 416 | 19 | **11.61** | 11.78 | 0.17 | **13.65** | 13.68 | 0.03 | **3.03** | 2.68 | -0.35 |
| 6 | 1 | 13 | 2 | 2 | **0.2** | 0.2 | 0.00 | **0.2** | 0.2 | 0.00 | **-10.25** | -10.75 | -0.500 | **57.3** | 57.2 | -0.1 | **71.2** | 69.7 | -1.5 | **389** | 418 | 29 | **5.39** | 6.43 | 1.04 | **8.29** | 8.84 | 0.55 | **2.62** | 2.63 | 0.01 |
| 52 | 1 | 20 | 2 | 2 | **1.1** | 1 | -0.10 | **0.8** | 0.1 | -0.70 | **-8.50** | -8.75 | -0.250 | **47.8** | 47.0 | -0.8 | **52.5** | 51 | -1.5 | **470** | 481 | 11 | **2.24** | 1.28 | -0.96 | **4.08** | 2.89 | -1.19 | **1.5** | 1.21 | -0.29 |
| 80 | 1 | 23 | 2 | 2 | **0.6** | 0.4 | -0.20 | **0.4** | 0.2 | -0.20 | **-5.125** | -5.125 | 0.000 | **47.1** | 47.3 | 0.2 | **54.5** | 54.3 | -0.2 | **488** | 494 | 6 | **2.4** | 2.21 | -0.19 | **3.8** | 3.55 | -0.25 | **1** | 0.94 | -0.06 |
| 40 | 1 | 18 | 2 | 2 | **0.8** | 0.8 | 0.00 | **0.2** | 0.1 | -0.10 | **-6.75** | -10.625 | -3.875 | **53.8** | 54.4 | 0.6 | **72** | 73.3 | **1.3** | **461** | 474 | 13 | **6.92** | 7.06 | 0.14 | **7.99** | 8.4 | 0.41 | **1.72** | 1.4 | -0.32 |
| 86 | 2 | 24 | 2 | 2 | **1** | 1 | 0.00 | **0.2** | 0.4 | 0.20 | **-19.75** | -9.25 | 10.500 | **56.4** | 58.4 | 2.0 | **74.2** | 76.7 | **2.5** | **395** | 404 | 9 | **8.16** | 8.35 | 0.19 | **8.66** | 8.8 | 0.14 | **3.04** | 2.92 | -0.12 |
| 63 | 2 | 21 | 2 | 2 | **0.7** | 0.7 | 0.00 | **0.1** | 0.1 | 0.00 | **-4.00** | -4.375 | -0.375 | **45.5** | 46.2 | 0.7 | **49.8** | 50.8 | 1.0 | **452** | 437 | -15 | **2.03** | 2.05 | 0.02 | **2.51** | 2.58 | 0.07 | **1.94** | 2.1 | 0.16 |
| 64 | 1 | 21 | 2 | 1 | **0.8** | 0.9 | 0.10 | **0.3** | 0.3 | 0.00 | **-10.875** | -11.375 | -0.500 | **54.8** | 55.1 | 0.3 | **65.6** | 67.2 | **1.6** | **359** | 380 | 21 | **2.39** | 2.45 | 0.06 | **4.55** | 4.06 | -0.49 | **2.85** | 2.89 | 0.04 |
| 47 | 1 | 19 | 2 | 1 | **1** | 1 | 0.00 | **0** | 0 | 0.00 | **-4.50** | -4.50 | 0.000 | **45.2** | 45.3 | 0.0 | **47.9** | 48.2 | 0.3 | **442** | 438 | -4 | **0.89** | 1.11 | 0.22 | **2.22** | 2.11 | -0.11 | **2.17** | 2.24 | 0.07 |
| 47 | 1 | 19 | 2 | 2 | **0.9** | 0.9 | 0.00 | **0** | 0 | 0.00 | **-3.875** | -3.875 | 0.000 | **45.4** | 46.0 | 0.6 | **50.1** | 51.4 | 1.3 | **416** | 422 | 6 | **2.02** | 2.08 | 0.06 | **2.72** | 2.8 | 0.08 | **2.69** | 2.56 | -0.13 |
| 97 | 2 | 27 | 2 | 2 | **0.9** | 0.6 | -0.30 | **0.2** | 0.4 | 0.20 | **-9.00** | -13.25 | -4.250 | **53.6** | 52.7 | -0.9 | **69.2** | 63.4 | -5.8 | **383** | 393 | 10 | **0** | 0 | 0 | **2.63** | 2.84 | 0.21 | **3.17** | 3.07 | -0.1 |
| 98 | 2 | 28 | 2 | 1 | **1** | 1 | 0.00 | **0.3** | 0 | -0.30 | **-7.625** | -7.625 | 0.000 | **46.2** | 46.6 | 0.4 | **52.5** | 53.9 | **1.4** | **437** | 437 | 0 | **2.48** | 2.6 | 0.12 | **4.83** | 4.63 | -0.2 | **2.1** | 2.27 | 0.17 |
| 98 | 2 | 28 | 2 | 2 | **0.8** | 0.8 | 0.00 | **0.3** | 0.4 | 0.10 | **-5.875** | -6.25 | -0.375 | **46.5** | 47.0 | 0.5 | **54.4** | 54.9 | 0.5 | **426** | 427 | 1 | **2.7** | 2.7 | 0 | **4.5** | 4.43 | -0.07 | **2.23** | 2.25 | 0.02 |
| 53 | 1 | 20 | 2 | 2 | **1** | 0.9 | -0.10 | **0.4** | 0.7 | 0.30 | **-10.375** | -12.125 | -1.750 | **50.3** | 50.2 | -0.1 | **60.1** | 59.8 | -0.3 | **473** | 468 | -5 | **3.81** | 3.42 | -0.39 | **5.93** | 6.08 | 0.15 | **1.43** | 1.54 | 0.11 |
| 41 | 1 | 18 | 2 | 2 | **0** | -0.1 | -0.10 | **0** | -0.1 | -0.10 | **0** | -0.125 | -0.125 | **44.8** | 45.4 | 0.6 | **52.6** | 51.3 | -1.3 | **431** | 432 | 1 | **2.1** | 1.65 | -0.45 | **2.52** | 2.38 | -0.14 | **2.7** | 2.35 | -0.35 |
| 54 | 1 | 20 | 2 | 2 | **1.2** | 0.9 | -0.30 | **0.2** | 0.3 | 0.10 | **-13.875** | -13.50 | 0.375 | **53.7** | 53.9 | 0.2 | **68.3** | 67.3 | -1.0 | **418** | 416 | -2 | **5.3** | 5.43 | 0.13 | **5.75** | 6.4 | 0.65 | **2.64** | 2.67 | 0.03 |
| 48 | 1 | 19 | 2 | 1 | **0.4** | 0.4 | 0.00 | **0.1** | 0.3 | 0.20 | **-2.00** | -3.125 | -1.125 | **44.6** | 45.0 | 0.4 | **49.5** | 48.2 | -1.3 | **491** | 489 | -2 | **1.34** | 1.19 | -0.15 | **2.77** | 2.72 | -0.05 | **0.98** | 1.01 | 0.03 |
| 96 | 1 | 26 | 2 | 1 | **0.7** | 0.7 | 0.00 | **0.1** | 0.3 | 0.20 | **-6.75** | -7.00 | -0.250 | **55.8** | 54.8 | -1.1 | **68.9** | 64.2 | -4.7 | **447** | 444 | -3 | **5.12** | 4.79 | -0.33 | **8.31** | 8.5 | 0.19 | **2.06** | 2.13 | 0.07 |
| 65 | 1 | 21 | 2 | 1 | **0.2** | 0.1 | -0.10 | **0** | 0 | 0.00 | **-1.75** | -1.50 | 0.250 | **45.2** | 45.2 | -0.0 | **46.8** | 47 | 0.2 | **517** | 523 | 6 | **0.49** | 0.43 | -0.06 | **0.47** | 0.2 | -0.27 | **0.61** | 0.52 | -0.09 |
| 87 | 2 | 24 | 2 | 1 | **1.1** | 1.4 | 0.30 | **0.4** | 0.7 | 0.30 | **-16.00** | -7.50 | 8.500 | **56.9** | 57.1 | 0.1 | **74.8** | 72.3 | -2.5 | **421** | 422 | 1 | **5.47** | 7.33 | 1.86 | **7.11** | 7.54 | 0.43 | **2.57** | 2.56 | -0.01 |
| 87 | 2 | 24 | 2 | 2 | **1** | 1.4 | 0.40 | **0.4** | 0.2 | -0.20 | **-15.00** | -12.75 | 2.250 | **52.2** | 51.9 | -0.4 | **66.8** | 63.9 | -2.9 | **449** | 445 | -4 | **3.21** | 4.52 | 1.31 | **5.94** | 6 | 0.06 | **2.02** | 2.09 | 0.07 |
| 55 | 1 | 20 | 2 | 1 | **1** | 1.2 | 0.20 | **0.7** | 0.4 | -0.30 | **-18.75** | -5.00 | 13.750 | **60.8** | 60.3 | -0.5 | **72.2** | 70.7 | -1.5 | **426** | 403 | -23 | **8.41** | 8.05 | -0.36 | **9.97** | 9.86 | -0.11 | **2.47** | 2.94 | 0.47 |
| 70 | 1 | 22 | 2 | 2 | **1.2** | 1.1 | -0.10 | **0.7** | 0.4 | -0.30 | **-11.00** | -7.75 | 3.250 | **58** | 59.3 | 1.3 | **77.7** | 76.6 | -1.1 | **425** | 436 | 11 | **8.43** | 8.97 | 0.54 | **9.38** | 9.38 | 0 | **2.51** | 2.27 | -0.24 |
| 147 | 1 | 29 | 2 | 1 | **1.2** | 1.7 | 0.50 | **0.5** | 0.3 | -0.20 | **-11.75** | -9.25 | 2.500 | **58.1** | 57.4 | -0.7 | **75.2** | 76 | 0.8 | **443** | 454 | 11 | **10.01** | 9.61 | -0.4 | **9.46** | 9.19 | -0.27 | **2.14** | 1.91 | -0.23 |
| 140 | 1 | 24 | 2 | 1 | **0.9** | 0.8 | -0.10 | **0.8** | 0 | -0.80 | **-15.00** | -5.00 | 10.000 | **51.5** | 49.3 | -2.2 | **67.3** | 62.5 | -4.8 | **432** | 427 | -5 | **3.68** | 3.41 | -0.27 | **5.44** | 5 | -0.44 | **2.36** | 2.45 | 0.09 |
| 123 | 2 | 17 | 2 | 1 | **0.8** | 0.8 | 0.00 | **0.1** | 0.2 | 0.10 | **-7.50** | -7.50 | 0.000 | **42.8** | 43 | 0.2 | **46.5** | 46.8 | 0.3 | **426** | 448 | 22 | **0** | 0 | 0 | **0.39** | 0.22 | -0.17 | **2.48** | 2.04 | -0.44 |
| 123 | 2 | 17 | 2 | 2 | **0.5** | 0.4 | -0.10 | **0.4** | 0.4 | 0.00 | **-8.25** | -7.00 | 1.250 | **48.5** | 47.7 | -0.8 | **64.3** | 61.8 | -2.5 | **401** | 425 | 24 | **2.82** | 2.51 | -0.31 | **4.32** | 3.27 | -1.05 | **2.97** | 2.49 | -0.48 |
| 143 | 1 | 26 | 2 | 2 | **0.9** | 0.8 | -0.10 | **0.6** | 0.7 | 0.10 | **-13** | -1 | 12.000 | **62.3** | 59.9 | -2.4 | **72.9** | 71.6 | -1.3 | **408** | 458 | 50 | **9.65** | 8.1 | -1.55 | **10.94** | 9.08 | -1.86 | **2.83** | 1.81 | -1.02 |
| 141 | 1 | 24 | 2 | 2 | **0.9** | 0.9 | 0.00 | **0.6** | 0.7 | 0.10 | **-19** | -17 | 2.000 | **54.5** | 52 | -2.5 | **67** | 61.2 | -5.8 | **413** | 406 | -7 | **6.52** | 4.85 | -1.67 | **9.89** | 9.12 | -0.77 | **2.73** | 2.87 | 0.14 |
| 27 | 1 | 24 | 2 | 1 | **0.9** | 0.7 | -0.20 | **0.5** | 0.4 | -0.10 | **-10.125** | -9.5 | 0.625 | **52.7** | 52.3 | -0.4 | **62.1** | 60.2 | -1.9 | **414** | 433 | 19 | **4.42** | 3.75 | -0.67 | **6.65** | 6.56 | -0.09 | **2.72** | 2.33 | -0.39 |
| 122 | 2 | 16 | 2 | 2 | **0.6** | 0.9 | 0.30 | **0.2** | 0.3 | 0.10 | **-7.25** | -7 | 0.250 | **48.9** | 50.1 | 1.2 | **63.6** | 62.2 | -1.4 | **433** | 425 | -8 | **4.66** | 3.98 | -0.68 | **5.58** | 4.66 | -0.92 | **2.34** | 2.5 | 0.16 |
| 117 | 1 | 15 | 2 | 2 | **0.4** | 0.8 | 0.40 | **0.4** | 0.2 | -0.20 | **-0.5** | -4.75 | -4.250 | **61.4** | 56.6 | -4.8 | **79.5** | 69.8 | -9.7 | **413** | 410 | -3 | **10.29** | 7.24 | -3.05 | **11.59** | 10.04 | -1.55 | **2.73** | 2.8 | 0.07 |
| 137 | 2 | 21 | 2 | 2 | **0.9** | 0.8 | -0.10 | **0.4** | 0.3 | -0.10 | **-3.75** | -9 | -5.250 | **47.8** | 46.3 | -1.5 | **59.4** | 56 | -3.4 | **407** | 399 | -8 | **2.97** | 2.64 | -0.33 | **4.99** | 4.6 | -0.39 | **2.86** | 3.01 | 0.15 |
| 150 | 2 | 32 | 2 | 1 | **1.7** | 1.4 | -0.30 | **0.5** | 0.7 | 0.20 | **-11.5** | -11.625 | -0.125 | **50.7** | 42.3 | -8.4 | **57.6** | 43 | -14.6 | **409** | 448 | 39 | **3.31** | 0 | -3.31 | **5.94** | 0 | -5.94 | **2.82** | 0.03 | -2.79 |
| 88 | 1 | 24 | 3 | 2 | **1** | 0.7 | -0.3 | **0.1** | 0 | -0.1 | **-3.25** | -3 | 0.25 | **42.5** | 42.3 | -0.2 | **45.2** | 44.2 | -1 | **489** | 484 | -5 | **0** | 0 | 0 | **0.26** | 0 | -0.26 | **1.01** | 1.16 | 0.15 |
| 105 | 2 | 32 | 3 | 1 | **1** | 0.8 | -0.2 | **0.3** | 0.1 | -0.2 | **-9.5** | -8.25 | 1.25 | **52.3** | 51.5 | -0.8 | **57** | 56 | -1 | **482** | 474 | -8 | **4.28** | 4.06 | -0.22 | **5.9** | 5.22 | -0.68 | **1.19** | 1.38 | 0.19 |
| 105 | 2 | 32 | 3 | 2 | **1.2** | 0.7 | -0.5 | **0.2** | 0.1 | -0.1 | **-7.875** | -7.25 | 0.625 | **49.5** | 48 | -1.5 | **53.9** | 52.8 | -1.1 | **485** | 462 | -23 | **2.95** | 2.62 | -0.33 | **5.11** | 4.78 | -0.33 | **1.12** | 1.7 | 0.58 |
| 108 | 2 | 39 | 3 | 1 | **0.6** | 0.5 | -0.1 | **0.6** | 0.4 | -0.2 | **0** | -2.00 | -2.00 | **42.7** | 43.0 | 0.3 | **53.6** | 51.7 | -1.9 | **474** | 454 | -20 | **1.08** | 1.81 | 0.73 | **2.25** | 3.12 | 0.87 | **1.25** | 1.9 | 0.65 |
| 28 | 1 | 16 | 3 | 1 | **0.7** | 0.7 | 0 | **0.1** | 0.1 | 0 | **-5.25** | -4.75 | 0.50 | **48** | 46.8 | -1.2 | **60.0** | 53.4 | -6.6 | **487** | 479 | -8 | **2.7** | 2.22 | -0.48 | **5.29** | 5.22 | -0.07 | **1.08** | 1.27 | 0.19 |
| 40 | 1 | 18 | 3 | 1 | **0.3** | 0.3 | 0 | **0.1** | 0.3 | 0.2 | **-8.125** | -8.50 | -0.375 | **49.8** | 51.2 | 1.4 | **65.8** | 65.8 | 0 | **476** | 476 | 0 | **4.48** | 4.7 | 0.22 | **6.98** | 7.37 | 0.39 | **1.35** | 1.34 | -0.01 |
| 92 | 2 | 25 | 3 | 1 | **0.3** | 0.3 | 0 | **0.2** | 0.1 | -0.1 | **-3.25** | -5.00 | -1.75 | **46.9** | 44.4 | -2.5 | **54.3** | 49.2 | -5.1 | **467** | 431 | -36 | **2.57** | 0.92 | -1.65 | **4.03** | 4.44 | 0.41 | **1.57** | 2.37 | 0.8 |
| 92 | 2 | 25 | 3 | 2 | **0.6** | 1.0 | 0.4 | **0.3** | 0.2 | -0.1 | **-8.625** | -6.50 | 2.125 | **49.5** | 47.5 | -2 | **58.2** | 54.7 | -3.5 | **450** | 425 | -25 | **3.04** | 2.44 | -0.6 | **5.7** | 6.14 | 0.44 | **2** | 2.5 | 0.5 |
| 82 | 1 | 23 | 3 | 2 | **0.5** | 0.6 | 0.1 | **0.1** | 0.1 | 0 | **-10.75** | -9.75 | 1.00 | **48.1** | 47.7 | -0.4 | **52.8** | 51.8 | -1 | **479** | 463 | -16 | **2.21** | 2.08 | -0.13 | **4.12** | 4.54 | 0.42 | **1.26** | 1.68 | 0.42 |
| 59 | 2 | 20 | 3 | 2 | **0.3** | 0 | -0.3 | **0.1** | 0 | -0.1 | **-2.75** | -1.375 | 1.375 | **48.9** | 46.2 | -2.7 | **57.2** | 51.8 | -5.4 | **452** | 446 | -6 | **2.91** | 2.25 | -0.66 | **5.44** | 5.84 | 0.4 | **1.85** | 2.07 | 0.22 |
| 38 | 1 | 17 | 3 | 2 | **0.2** | 0.2 | 0 | **0** | 0 | 0 | **-4.875** | -7.50 | -2.625 | **46.9** | 47.9 | 1 | **58.1** | 56.8 | -1.3 | **537** | 513 | -24 | **2.43** | 2.52 | 0.09 | **3.51** | 4.69 | 1.18 | **0.32** | 0.84 | 0.52 |
| 44 | 1 | 18 | 3 | 2 | **0.7** | 0.6 | -0.1 | **0** | -0.1 | -0.1 | **-2.75** | -2.75 | 0 | **43.3** | 42.7 | -0.6 | **44.1** | 44.1 | 0 | **492** | 480 | -12 | **0** | 0 | 0 | **0** | 0.2 | 0.2 | **0.97** | 0.97 | 0 |
| 66 | 1 | 21 | 3 | 2 | **0.8** | 0.8 | 0 | **0.1** | 0.1 | 0 | **-8.50** | -7.375 | 1.125 | **44.3** | 43.8 | -0.5 | **46.6** | 44.8 | -1.8 | **471** | 468 | -3 | **0.42** | 0 | -0.42 | **1.9** | 1.37 | -0.53 | **1.57** | 1.53 | -0.04 |
| 104 | 1 | 32 | 3 | 2 | **1.2** | 1.2 | 0 | **0.3** | 0.1 | -0.2 | **-7.75** | -7.00 | 0.75 | **43.7** | 43 | -0.7 | **52.5** | 51.7 | -0.8 | **475** | 466 | -12 | **2.3** | 2.02 | -0.28 | **2.52** | 2.53 | 0.01 | **1.35** | 1.58 | 0.23 |
| 101 | 2 | 29 | 3 | 2 | **1.4** | 1.1 | -0.3 | **0.3** | 0.1 | -0.2 | **-7.375** | -8.50 | -1.125 | **50.3** | 50 | -0.3 | **55.4** | 52.6 | -2.8 | **466** | 455 | -11 | **2.98** | 2.66 | -0.32 | **4.68** | 5.15 | 0.47 | **1.61** | 1.87 | 0.26 |
| 71 | 2 | 22 | 3 | 1 | **1.4** | 1.4 | 0 | **0** | 0 | 0 | **-7.125** | -9.125 | -2 | **46.5** | 45.4 | -1.1 | **55.5** | 54.2 | -1.3 | **459** | 438 | -21 | **2.3** | 1.96 | -0.34 | **2.57** | 2.74 | 0.17 | **1.43** | 2.23 | 0.8 |
| 49 | 1 | 19 | 3 | 1 | **0.4** | 0.2 | -0.2 | **0.1** | 0 | -0.1 | **-6.00** | -7.625 | -1.625 | **45** | 44.7 | -0.3 | **47.6** | 47.7 | 0.1 | **474** | 489 | 15 | **0.53** | 0.31 | -0.22 | **0** | 0 | 0 | **1.4** | 1.01 | -0.39 |
| 72 | 1 | 22 | 3 | 1 | **0.8** | 0.8 | 0 | **0.2** | 0.3 | 0.1 | **-5.00** | -7.00 | -2 | **45.7** | 46.2 | 0.5 | **58.7** | 58.4 | -0.3 | **479** | 454 | -25 | **2.43** | 3.54 | 1.11 | **5.41** | 6.97 | 1.56 | **1.27** | 1.89 | 0.62 |
| 106 | 1 | 34 | 3 | 1 | **1.2** | 0.8 | -0.4 | **0.3** | 0.1 | -0.2 | **-13.625** | -13.00 | 0.625 | **53.4** | 54.4 | 1 | **62.6** | 61.6 | -1 | **468** | 475 | 7 | **5.43** | 5.86 | 0.43 | **7.89** | 8.13 | 0.24 | **1.56** | 1.38 | -0.18 |
| 70 | 1 | 22 | 3 | 1 | **0.4** | 0.4 | 0 | **0.1** | 0.1 | 0 | **-7.75** | -6.75 | 1 | **46.3** | 45.7 | -0.6 | **52.3** | 52.4 | 0.1 | **480** | 483 | 3 | **2.2** | 2 | -0.2 | **2.89** | 2.84 | -0.05 | **1.26** | 1.17 | -0.09 |
| 23 | 1 | 16 | 3 | 2 | **1** | 1.7 | 0.7 | **0.5** | 0.2 | -0.3 | **-10.50** | -15.00 | -4.5 | **51.3** | 51.2 | -0.1 | **58.3** | 56 | -2.3 | **501** | 469 | -32 | **3.69** | 2.88 | -0.81 | **6.76** | 8.22 | 1.46 | **0.83** | 1.53 | 0.7 |
| 56 | 1 | 20 | 3 | 2 | **1.4** | 1.4 | 0 | **0** | 0.1 | 0.1 | **-7.00** | -6.25 | 0.75 | **43.8** | 42.1 | -1.7 | **46.7** | 44.3 | -2.4 | **504** | 499 | -5 | **0.11** | 0 | -0.11 | **0.06** | 0.27 | 0.21 | **0.8** | 0.86 | 0.06 |
| 29 | 1 | 17 | 3 | 2 | **0.4** | 0.4 | 0 | **0.4** | 0.2 | -0.2 | **-8.00** | -4.50 | 3.5 | **50.1** | 49 | -1.1 | **68.2** | 63.9 | -4.3 | **485** | 444 | -41 | **4.97** | 3.91 | -1.06 | **6.9** | 6.84 | -0.06 | **1.13** | 2.11 | 0.98 |
| 93 | 1 | 26 | 3 | 2 | **1.2** | 1 | -0.2 | **0** | 0 | 0 | **-7.75** | -8.00 | -0.25 | **44.3** | 43.2 | -1.1 | **51.5** | 49.9 | -1.6 | **470** | 447 | -23 | **1.54** | 0.57 | -0.97 | **2.92** | 4.59 | 1.67 | **1.51** | 2.06 | 0.55 |
| 120 | 1 | 16 | 3 | 2 | **0.3** | 0.3 | 0 | **0.2** | 0.1 | -0.1 | **-3.00** | -1.50 | 1.5 | **43** | 42.8 | -0.2 | **46.1** | 45.5 | -0.6 | **582** | 564 | -18 | **0.03** | 0.11 | 0.08 | **1.17** | 1.79 | 0.62 | **0** | 0 | 0 |
| 121 | 1 | 16 | 3 | 1 | **0.4** | 0.3 | -0.1 | **0.4** | 0.2 | -0.2 | **-4.75** | -7.125 | -2.375 | **45.4** | 45.3 | -0.1 | **50.9** | 49.7 | -1.2 | **497** | 486 | -11 | **2.11** | 2.02 | -0.09 | **3.42** | 4.21 | 0.79 | **0.77** | 1.07 | 0.3 |
| 140 | 1 | 24 | 3 | 2 | **0.4** | 0.6 | 0.2 | **0.1** | 0 | -0.1 | **-7.125** | -5.00 | 2.125 | **43.9** | 43 | -0.9 | **47.3** | 45.7 | -1.6 | **487** | 468 | -19 | **0.21** | 0 | -0.21 | **0.03** | 0.83 | 0.8 | **1.06** | 1.56 | 0.5 |
| 146 | 2 | 28 | 3 | 2 | **0.8** | 0.7 | -0.1 | **0.1** | 0.1 | 0 | **-8.25** | -7.25 | 1 | **45** | 45.1 | 0.1 | **47.4** | 46.5 | -0.9 | **514** | 501 | -13 | **0.73** | 0.67 | -0.06 | **1.27** | 1.22 | -0.05 | **0.65** | 0.83 | 0.18 |
| 148 | 2 | 29 | 3 | 2 | **0.5** | 0.1 | -0.4 | **0.4** | 0.1 | -0.3 | **-6.25** | -5.50 | 0.75 | **50** | 48.4 | -1.6 | **60.6** | 56.3 | -4.3 | **472** | 425 | -47 | **3.88** | 2.86 | -1.02 | **7.67** | 8.13 | 0.46 | **2.59** | 2.49 | -0.1 |
| 142 | 2 | 25 | 3 | 1 | **1.4** | 1.4 | 0 | **0.4** | 0.2 | -0.2 | **-11.75** | -7.75 | 4 | **48.8** | 46.7 | -2.1 | **56.2** | 51 | -5.2 | **483** | 470 | -13 | **2.55** | 1.97 | -0.58 | **3.73** | 3.43 | -0.3 | **1.18** | 1.5 | 0.32 |
| 122 | 2 | 16 | 3 | 1 | **0.8** | 1 | 0.2 | **0.1** | 0.1 | 0 | **-8.75** | -8.75 | 0 | **44.1** | 43.5 | -0.6 | **46.5** | 44.9 | -1.6 | **449** | 438 | -11 | **0.43** | 0 | -0.43 | **0.54** | 0.92 | 0.38 | **2.01** | 2.24 | 0.23 |
| 135 | 1 | 20 | 3 | 2 | **0.8** | 0.6 | -0.2 | **0.4** | 0.4 | 0 | **7.25** | -0.75 | -8 | **52.2** | 52.4 | 0.2 | **64** | 62.1 | -1.9 | **450** | 432 | -18 | **3.54** | 5.44 | 1.9 | **4.05** | 6.14 | 2.09 | **1.99** | 2.35 | 0.36 |
| 117 | 1 | 15 | 3 | 1 | **0.4** | 0.8 | 0.4 | **0.2** | 0.1 | -0.1 | **-1.125** | -2.375 | -1.25 | **41.4** | 41 | -0.4 | **51.2** | 50.5 | -0.7 | **506** | 496 | -10 | **1.47** | 1.33 | -0.14 | **2.35** | 2.46 | 0.11 | **0.76** | 0.91 | 0.15 |
| 118 | 1 | 15 | 3 | 2 | **0.9** | 0.5 | -0.4 | **0.1** | 0 | -0.1 | **-6** | -6.5 | -0.5 | **45.8** | 45.9 | 0.1 | **47.2** | 46.5 | -0.7 | **546** | 527 | -19 | **0.95** | 1.29 | 0.34 | **2.41** | 2.87 | 0.46 | **0.19** | 0.46 | 0.27 |
| 134 | 2 | 19 | 3 | 1 | **1** | 1.1 | 0.1 | **0.4** | 0.4 | 0 | **-10.125** | -5.5 | 4.625 | **54.9** | 55.8 | 0.9 | **70.9** | 68.6 | -2.3 | **473** | 439 | -34 | **2.96** | 7.43 | 4.47 | **5.21** | 8.81 | 3.6 | **1.43** | 2.22 | 0.79 |
| 134 | 2 | 19 | 3 | 2 | **0.9** | 0.7 | -0.2 | **0.5** | 0.3 | -0.2 | **-5.75** | -3.5 | 2.25 | **48.6** | 48.3 | -0.3 | **56.4** | 54.2 | -2.2 | **494** | 483 | -11 | **2.7** | 2.58 | -0.12 | **4.47** | 5.51 | 1.04 | **0.94** | 1.18 | 0.24 |
| 139 | 2 | 23 | 3 | 1 | **1** | 0.9 | -0.1 | **0.5** | 0.3 | -0.2 | **-7.25** | -7.25 | 0 | **50.6** | 50.5 | -0.1 | **57.8** | 55 | -2.8 | **455** | 445 | -10 | **4.33** | 3.53 | -0.8 | **6.65** | 6.48 | -0.17 | **1.86** | 2.1 | 0.24 |
| 139 | 2 | 23 | 3 | 2 | **0.8** | 0.3 | -0.5 | **0.5** | 0.2 | -0.3 | **-6.25** | -6.875 | -0.625 | **50.5** | 50.1 | -0.4 | **59.6** | 56 | -3.6 | **456** | 448 | -8 | **4.24** | 3.81 | -0.43 | **7.03** | 6.78 | -0.25 | **1.84** | 2.03 | 0.19 |
| 153 | 2 | 35 | 3 | 2 | **0.8** | 0.9 | 0.1 | **0.3** | 0.4 | 0.1 | **-11.375** | -11.375 | 0 | **53.6** | 51.3 | -2.3 | **68.6** | 60.6 | -8 | **451** | 419 | -32 | **6.93** | 4.76 | -2.17 | **8.19** | 8.62 | 0.43 | **1.98** | 2.61 | 0.63 |
| 116 | 1 | 15 | 4 | 2 | **0.8** | 0.5 | -0.3 | **0** | 0 | 0 | **-2.875** | -3.25 | -0.375 | **47.8** | 46.8 | -1 | **50.3** | 51.9 | 1.6 | **578** | 578 | 0 | **2** | 0.69 | -1.31 | **2.4** | 2.32 | -0.08 | **0** | 0 | 0 |
| 32 | 1 | 17 | 4 | 1 | **0.4** | 0.4 | 0 | **0.1** | 0.1 | 0 | **-6.75** | -4.50 | 2.25 | **43.2** | 42.8 | -0.4 | **48.4** | 47.9 | -0.5 | **506** | 500 | -6 | **0.33** | 0.25 | -0.08 | **1.8** | 1.99 | 0.19 | **0.77** | 0.91 | 0.14 |
| 45 | 1 | 18 | 4 | 1 | **0.8** | 1.1 | 0.3 | **0** | 0 | 0 | **-6.75** | -7.00 | -0.25 | **43.4** | 43.7 | 0.3 | **47.1** | 45.8 | -1.3 | **476** | 463 | -13 | **0.72** | 0.65 | -0.07 | **2.58** | 3.12 | 0.54 | **1.33** | 1.66 | 0.33 |
| 57 | 1 | 20 | 4 | 1 | **0.5** | 0.5 | 0 | **0.3** | 0.4 | 0.1 | **-10.625** | -11.875 | -1.25 | **57.8** | 57.2 | -0.6 | **75.1** | 72.8 | -2.3 | **473** | 499 | 26 | **5.95** | 5.07 | -0.88 | **6.03** | 5.8 | -0.23 | **1.43** | 0.87 | -0.56 |
| 42 | 1 | 18 | 4 | 1 | **0.8** | 0.3 | -0.5 | **0.1** | 0.1 | 0 | **-4.875** | -5.25 | -0.375 | **45.1** | 45.2 | 0.1 | **54.0** | 52.3 | -1.7 | **477** | 477 | 0 | **2.15** | 1.51 | -0.64 | **1.91** | 2.85 | 0.94 | **0.55** | 1.32 | 0.77 |
| 33 | 1 | 17 | 4 | 1 | **0.7** | 0.3 | -0.4 | **0.3** | 0 | -0.3 | **-5.75** | -3.125 | 2.625 | **42.6** | 42 | -0.6 | **49.1** | 45.1 | -4 | **488** | 473 | -15 | **1.5** | 0.04 | -1.46 | **2.5** | 2.47 | -0.03 | **1.97** | 1.42 | -0.55 |
| 33 | 1 | 17 | 4 | 2 | **0.8** | 0.8 | 0 | **0.4** | 0 | -0.4 | **-9.00** | -4.875 | 4.125 | **44.9** | 45.8 | 0.9 | **54.0** | 55.5 | 1.5 | **472** | 478 | 6 | **3** | 2.7 | -0.3 | **4** | 3.7 | -0.3 | **1.72** | 1.3 | -0.42 |
| 4 | 1 | 11 | 4 | 1 | **1** | 1.1 | 0.1 | **0.1** | 0.1 | 0 | **-4.125** | -10.00 | -5.875 | **53** | 53.4 | 0.4 | **63.7** | 61 | -2.7 | **474** | 412 | -62 | **4.18** | 5.08 | 0.9 | **6.13** | 8.2 | 2.07 | **1.31** | 2.77 | 1.46 |
| 4 | 1 | 11 | 4 | 2 | **0.9** | 1.1 | 0.2 | **0.2** | 0 | -0.2 | **-3.25** | -6.25 | -3 | **53.5** | 51.5 | -2 | **60.3** | 57.8 | -2.5 | **484** | 446 | -38 | **5.11** | 2.82 | -2.29 | **5.4** | 5.56 | 0.16 | **1.15** | 2.07 | 0.92 |
| 9 | 1 | 14 | 4 | 1 | **0.9** | 1 | 0.1 | **0.2** | 0.1 | -0.1 | **-6.125** | -5.00 | 1.125 | **47.6** | 47.4 | -0.2 | **53.1** | 52.4 | -0.7 | **509** | 496 | -13 | **2.17** | 1.76 | -0.41 | **2.51** | 2.54 | 0.03 | **0.73** | 0.9 | 0.17 |
| 9 | 1 | 14 | 4 | 2 | **0.4** | 0.1 | -0.3 | **0.2** | 0.1 | -0.1 | **-4.25** | -1.50 | 2.75 | **49.3** | 48.2 | -1.1 | **58.1** | 54.5 | -3.6 | **491** | 489 | -2 | **2.64** | 2.32 | -0.32 | **3.88** | 2.94 | -0.94 | **0.98** | 1.03 | 0.05 |
| 34 | 1 | 17 | 4 | 1 | **0.9** | 0.8 | -0.1 | **0.1** | 0 | -0.1 | **-5.75** | -7.5 | -1.75 | **45.2** | 45.6 | 0.4 | **51.7** | 50.3 | -1.4 | **509** | 499 | -10 | **2.18** | 2.06 | -0.12 | **3.14** | 3.47 | 0.33 | **0.73** | 0.87 | 0.14 |
| 17 | 1 | 15 | 4 | 2 | **0.3** | 0.1 | -0.2 | **0** | 0 | 0 | **-1.625** | -1.50 | 0.125 | **46.2** | 46.4 | 0.2 | **48.2** | 48.6 | 0.4 | **482** | 478 | -4 | **1.25** | 1.49 | 0.24 | **1.71** | 2.09 | 0.38 | **1.21** | 1.29 | 0.08 |
| 121 | 1 | 16 | 4 | 2 | **0.7** | 0.6 | -0.1 | **0.4** | 0.3 | -0.1 | **-4.50** | -6.00 | -1.5 | **47.1** | 46.4 | -0.7 | **54.4** | 54.6 | 0.2 | **471** | 473 | 2 | **2.46** | 2.46 | 0 | **6.47** | 6.04 | -0.43 | **1.48** | 1.41 | -0.07 |
| 110 | 1 | 10 | 4 | 1 | **0.6** | 0.5 | -0.1 | **0** | 0 | 0 | **-4.00** | -2.25 | 1.75 | **44.5** | 44.5 | 0 | **50.1** | 50.9 | 0.8 | **477** | 463 | -14 | **0.42** | 0.52 | 0.1 | **0.71** | 1.38 | 0.67 | **1.33** | 1.66 | 0.33 |
| 110 | 1 | 10 | 4 | 2 | **0.4** | 0.1 | -0.3 | **0.2** | 0.1 | -0.1 | **-5.375** | -0.25 | 5.125 | **47.3** | 47.3 | 0 | **54.3** | 55.2 | 0.9 | **466** | 460 | -6 | **1.44** | 1.44 | 0 | **2.31** | 2.36 | 0.05 | **1.6** | 1.74 | 0.14 |
| 125 | 1 | 18 | 4 | 1 | **0.2** | -0.2 | -0.4 | **0.1** | -0.2 | -0.3 | **0.5** | 0 | -0.5 | **44.1** | 44.4 | 0.3 | **50.3** | 50.4 | 0.1 | **503** | 498 | -5 | **0.46** | 0.54 | 0.08 | **2.17** | 2.23 | 0.06 | **0.8** | 0.89 | 0.09 |
| 125 | 1 | 18 | 4 | 1 | **0.7** | 0.7 | 0 | **0.3** | 0.7 | 0.4 | **-7.75** | -7.75 | 0 | **59** | 58.7 | -0.3 | **74.2** | 73.7 | -0.5 | **459** | 450 | -9 | **8.5** | 8.04 | -0.46 | **9** | 9.09 | 0.09 | **1.76** | 2 | 0.24 |
| 130 | 1 | 19 | 4 | 1 | **1.2** | 0.8 | -0.4 | **0.1** | 0 | -0.1 | **-4.375** | -4.25 | 0.125 | **46.1** | 46.2 | 0.1 | **48.9** | 49 | 0.1 | **507** | 485 | -22 | **1.38** | 1.65 | 0.27 | **2.25** | 2.27 | 0.02 | **0.76** | 1.13 | 0.37 |
| 130 | 1 | 19 | 4 | 2 | **0.5** | 0.7 | 0.2 | **0.2** | 0.3 | 0.1 | **-1.375** | -7.75 | -6.375 | **49.3** | 49.8 | 0.5 | **61.1** | 62.1 | 1 | **467** | 444 | -23 | **3.59** | 3.82 | 0.23 | **6.2** | 5.72 | -0.48 | **1.58** | 2.11 | 0.53 |
| 144 | 1 | 26 | 4 | 1 | **1.7** | 0.8 | -0.9 | **0** | 0 | 0 | **-8** | -7.875 | 0.125 | **45.4** | 45.6 | 0.2 | **47** | 47.4 | 0.4 | **482** | 481 | -1 | **1.37** | 0.98 | -0.39 | **2.13** | 2.4 | 0.27 | **1.2** | 1.22 | 0.02 |
| 113 | 1 | 14 | 4 | 1 | **0.8** | 0.7 | -0.1 | **0.1** | 0 | -0.1 | **-7.375** | -7.375 | 0 | **45.6** | 45.1 | -0.5 | **45.6** | 47.3 | 1.7 | **494** | 492 | -2 | **0.3** | 0.23 | -0.07 | **0.45** | 1.87 | 1.42 | **0.93** | 0.97 | 0.04 |
| 131 | 2 | 19 | 4 | 1 | **1** | 0.8 | -0.2 | **0** | 0 | 0 | **-4.25** | -4.75 | -0.5 | **41.9** | 42 | 0.1 | **42.5** | 42.7 | 0.2 | **485** | 482 | -3 | **0** | 0 | 0 | **0** | 0 | 0 | **1.11** | 1.19 | 0.08 |
| 131 | 2 | 19 | 4 | 2 | **0.5** | 0.3 | -0.2 | **0** | 0.1 | 0.1 | **-5.125** | -4 | 1.125 | **43.6** | 44.3 | 0.7 | **48.5** | 49.5 | 1 | **469** | 460 | -9 | **0.55** | 0.83 | 0.28 | **2.13** | 2.37 | 0.24 | **1.51** | 1.74 | 0.23 |
| 151 | 2 | 33 | 4 | 1 | **0.7** | 0.7 | 0 | **0.7** | 0.5 | -0.2 | **-6.5** | -6.5 | 0 | **47.1** | 48.3 | 1.2 | **55.9** | 56.2 | 0.3 | **488** | 506 | 18 | **2.63** | 2.98 | 0.35 | **4.24** | 4.73 | 0.49 | **1.05** | 0.76 | -0.29 |
| 126 | 1 | 18 | 4 | 1 | **1.4** | 1.2 | -0.2 | **0.1** | 0.1 | 0 | **-8.625** | -10.625 | -2 | **43.8** | 42.3 | -1.5 | **43.8** | 43.5 | -0.3 | **565** | 564 | -1 | **0** | 0 | 0 | **0** | 00 | 0 | **0** | 0 | 0 |
| 126 | 1 | 18 | 4 | 2 | **1.2** | 0.8 | -0.4 | **0.1** | 0.1 | 0 | **-5.875** | -7.875 | -2 | **42.4** | 42.3 | -0.1 | **43.7** | 44 | 0.3 | **565** | 565 | 0 | **0** | 0 | 0 | **0** | 0 | 0 | **0** | 0 | 0 |
| 153 | 2 | 35 | 4 | 1 | **0.5** | 0.5 | 0 | **0.1** | 0 | -0.1 | **-5** | -3.125 | 1.875 | **44.6** | 43.4 | -1.2 | **47.9** | 45.4 | -2.5 | **491** | 458 | -33 | **0.65** | 0 | -0.65 | **2.34** | 2.9 | 0.56 | **0.98** | 1.81 | 0.83 |
| 132 | 1 | 19 | 4 | 1 | **0.2** | 0.2 | 0 | **0** | 0 | 0 | **-0.75** | -1.5 | -0.75 | **42.5** | 42.3 | -0.2 | **45.9** | 44.3 | -1.6 | **517** | 509 | -8 | **0** | 0 | 0 | **0.64** | 0.73 | 0.09 | **0.61** | 0.73 | 0.12 |
| 132 | 1 | 19 | 4 | 2 | **0.3** | 0.3 | 0 | **0.3** | 0.2 | -0.1 | **-4.75** | -4.75 | 0 | **46.2** | 46.6 | 0.4 | **55.7** | 56.1 | 0.4 | **493** | 487 | -6 | **2.27** | 2.35 | 0.08 | **2.91** | 3.25 | 0.34 | **0.95** | 1.08 | 0.13 |
| 124 | 1 | 17 | 4 | 1 | **0.9** | 0.8 | -0.1 | **0.4** | 0.3 | -0.1 | **-3.5** | -3.375 | 0.125 | **53.0** | 51.6 | -1.4 | **67.0** | 61.7 | -5.3 | **455** | 419 | -36 | **6.54** | 5.38 | -1.16 | **7.7** | 8.28 | 0.58 | **1.88** | 2.61 | 0.73 |
| 124 | 1 | 17 | 4 | 2 | **0.8** | 0.5 | -0.3 | **0** | 0 | 0 | **-2.75** | -2.625 | 0.125 | **45.1** | 45.2 | 0.1 | **46.8** | 46.6 | -0.2 | **514** | 509 | -5 | **0.7** | 0.62 | -0.08 | **0.99** | 1.14 | 0.15 | **0.65** | 0.72 | 0.07 |
| 109 | 1 | 43 | 5 | 1 | **0.2** | 0.5 | 0.3 | **0** | 0 | 0 | **-1.75** | -2.75 | -1.00 | **49.1** | 49.2 | 0.1 | **51.0** | 52.2 | 1.2 | **490** | 480 | -10 | **2.34** | 2.44 | 0.1 | **2.89** | 2.93 | 0.04 | **0.99** | 1.25 | 0.26 |
| 109 | 1 | 43 | 5 | 2 | **1.0** | 1.0 | 0 | **0** | 0.2 | 0.2 | **-11.25** | -11.75 | -0.50 | **49.7** | 49.4 | -0.3 | **55.4** | 55.5 | 0.1 | **478** | 471 | -7 | **2.57** | 2.53 | -0.04 | **4.2** | 4.17 | -0.03 | **1.29** | 1.46 | 0.17 |
| 73 | 1 | 22 | 5 | 1 | **0.8** | 0.8 | 0 | **0.2** | 0.2 | 0 | **-9.00** | -7.50 | 1.50 | **48.6** | 48.4 | -0.2 | **58.8** | 58.4 | -0.4 | **479** | 473 | -6 | **2.72** | 2.63 | -0.09 | **5.13** | 5.13 | 0 | **1.28** | 1.43 | 0.15 |
| 73 | 1 | 22 | 5 | 2 | **0.8** | 0.9 | 0.1 | **0** | 0.1 | 0.1 | **-5.00** | -6.875 | -1.875 | **44.3** | 44 | -0.3 | **50.2** | 49.5 | -0.7 | **505** | 495 | -10 | **1.09** | 1.16 | 0.07 | **2.71** | 2.76 | 0.05 | **0.78** | 0.93 | 0.15 |
| 96 | 1 | 26 | 5 | 2 | **0.3** | 0.3 | 0 | **0.1** | 0 | -0.1 | **-4.25** | -4.50 | -0.25 | **51.3** | 50.2 | -1.1 | **63.9** | 60.5 | -3.4 | **477** | 479 | 2 | **3.59** | 2.86 | -0.73 | **6.19** | 5.93 | -0.26 | **1.31** | 1.26 | -0.05 |
| 83 | 1 | 23 | 5 | 1 | **1.4** | 1.1 | -0.3 | **0** | 0 | 0 | **-5.625** | -5.625 | 0 | **44** | 43.7 | -0.3 | **48.6** | 48.0 | -0.6 | **474** | 473 | -1 | **0.53** | 0.64 | 0.11 | **2.04** | 1.53 | -0.51 | **1.4** | 1.43 | 0.03 |
| 83 | 1 | 23 | 5 | 2 | **1.4** | 1.1 | -0.3 | **0** | 0 | 0 | **-5.875** | -6.625 | -0.75 | **44.2** | 44.5 | 0.3 | **47.9** | 47.5 | -0.4 | **487** | 487 | 0 | **0.57** | 0.58 | 0.01 | **1.3** | 1.36 | 0.06 | **1.06** | 1.06 | 0 |
| 94 | 2 | 26 | 5 | 1 | **1.7** | 1.4 | -0.3 | **0.1** | 0.1 | 0 | **-12.25** | -12.75 | -0.50 | **47.5** | 47.4 | -0.1 | **50.1** | 49.1 | -1 | **504** | 511 | 7 | **2.05** | 1.87 | -0.18 | **3.1** | 3.01 | -0.09 | **0.8** | 0.7 | -0.1 |
| 99 | 1 | 28 | 5 | 1 | **1** | 0.8 | -0.2 | **0.4** | 0.3 | -0.1 | **-4.375** | -3.25 | 1.125 | **47.9** | 47.7 | -0.2 | **56.5** | 56 | -0.5 | **453** | 460 | 7 | **2.66** | 2.5 | -0.16 | **3.57** | 3.8 | 0.23 | **1.92** | 1.8 | -0.12 |
| 84 | 1 | 23 | 5 | 1 | **1** | 0.8 | -0.2 | **0.4** | 0.4 | 0 | **-8.375** | -8.00 | 0.375 | **46.5** | 46 | -0.5 | **55** | 51.6 | -3.4 | **462** | 455 | -7 | **2.2** | 2 | -0.2 | **4.17** | 4.57 | 0.4 | **1.78** | 1.07 | -0.71 |
| 84 | 1 | 23 | 5 | 2 | **0.8** | 0.8 | 0 | **0.2** | 0 | -0.2 | **-6.875** | -5.375 | 1.5 | **42.9** | 41.2 | -1.7 | **47.1** | 45.7 | -1.4 | **482** | 456 | -26 | **0** | 0 | 0 | **1.78** | 2.15 | 0.37 | **1.19** | 1.84 | 0.65 |
| 24 | 1 | 16 | 5 | 2 | **0.3** | 0.1 | -0.2 | **0** | 0 | 0 | **-2.375** | 0 | 2.375 | **46.4** | 43.5 | -2.9 | **52.3** | 49 | -3.3 | **472** | 406 | -66 | **2.12** | 0.63 | -1.49 | **2.69** | 4.68 | 1.99 | **1.45** | 2.87 | 1.42 |
| 10 | 1 | 14 | 5 | 2 | **0.7** | 0.7 | 0 | **0.7** | 0.3 | -0.4 | **-8.50** | -8.375 | 0.125 | **48.8** | 49 | 0.2 | **58.2** | 57.3 | -0.9 | **497** | 496 | -1 | **3.58** | 4.38 | 0.8 | **5.87** | 5.81 | -0.06 | **0.89** | 0.9 | 0.01 |
| 50 | 1 | 19 | 5 | 2 | **0.8** | 0.8 | 0 | **0.4** | 0.4 | 0 | **-6.25** | -5.00 | 1.25 | **47.8** | 47.2 | -0.6 | **59.6** | 57.9 | -1.7 | **459** | 453 | -6 | **2.58** | 2.4 | -0.18 | **4.12** | 4.36 | 0.24 | **1.76** | 1.9 | 0.14 |
| 85 | 1 | 23 | 5 | 1 | **1.2** | 1.0 | -0.2 | **0.2** | 0.4 | 0.2 | **-4.00** | -3.625 | 0.375 | **46.4** | 46.8 | 0.4 | **62.5** | 62.1 | -0.4 | **462** | 466 | 4 | **3.52** | 6.75 | 3.23 | **5.39** | 7.53 | 2.14 | **1.7** | 1.6 | -0.1 |
| 95 | 1 | 26 | 5 | 1 | **0.4** | 0.4 | 0 | **0.4** | 0 | -0.4 | **-4.50** | -2.125 | 2.375 | **46.5** | 45.1 | -1.4 | **55.8** | 51.5 | -4.3 | **460** | 463 | 3 | **2.18** | 1.44 | -0.74 | **2.79** | 2.83 | 0.04 | **1.75** | 1.67 | -0.08 |
| 95 | 1 | 26 | 5 | 2 | **1.3** | 0.6 | -0.7 | **0.7** | 0.2 | -0.5 | **-4.00** | -2.125 | 1.875 | **48.4** | 47.3 | -1.1 | **60.4** | 58.9 | -1.5 | **470** | 456 | -14 | **2.78** | 2.46 | -0.32 | **4.3** | 4.29 | -0.01 | **1.49** | 1.84 | 0.35 |
| 136 | 2 | 20 | 5 | 1 | **0.9** | 0.8 | -0.1 | **0** | 0 | 0 | **-6.375** | -2.75 | 3.625 | **43.5** | 42.6 | -0.9 | **46.7** | 46.1 | -0.6 | **502** | 495 | -7 | **0.39** | 0 | -0.39 | **2.12** | 2.2 | 0.08 | **0.82** | 0.9 | 0.08 |
| 63 | 2 | 21 | 5 | 1 | **0.5** | 0.7 | 0.2 | **0** | 0 | 0 | **-3.25** | -4.00 | -0.75 | **45.1** | 45.1 | 0 | **46.5** | 46.8 | 0.3 | **472** | 478 | 6 | **0.63** | 0.63 | 0 | **1.43** | 1.58 | 0.15 | **1.44** | 1.6 | 0.16 |
| 145 | 2 | 26 | 5 | 1 | **0.8** | 0.8 | 0 | **0.4** | 0.4 | 0 | **-11.75** | -10.25 | 1.5 | **52.7** | 52.3 | -0.4 | **63.1** | 61 | -2.1 | **468** | 476 | 8 | **6.18** | 5.61 | -0.57 | **8.03** | 7.56 | -0.47 | **1.55** | 1.36 | -0.19 |
| 81 | 1 | 23 | 5 | 1 | **1.4** | 1 | -0.4 | **0.1** | 0 | -0.1 | **-10.75** | -7.5 | 3.25 | **43.8** | 45.4 | 1.6 | **51.6** | 51.3 | -0.3 | **510** | 497 | -13 | **2.18** | 2.13 | -0.05 | **2.97** | 2.97 | 0 | **0.72** | 0.89 | 0.17 |
| 30 | 1 | 17 | 5 | 1 | **0.1** | 0.1 | 0 | **0.8** | 0.5 | -0.3 | **-3.5** | -3.75 | -0.25 | **45.7** | 45.5 | -0.2 | **56.2** | 54.9 | -1.3 | **477** | 482 | 5 | **2.24** | 2.18 | -0.06 | **3.58** | 3.71 | 0.13 | **1.33** | 1.19 | -0.14 |
| 13 | 1 | 15 | 5 | 1 | **0.5** | 0.2 | -0.3 | **0** | 0.1 | 0.1 | **-0.75** | -6.25 | -5.5 | **48.6** | 48.8 | 0.2 | **54** | 54 | 0 | **498** | 487 | -11 | **2.56** | 2.43 | -0.13 | **4.06** | 4.6 | 0.54 | **0.88** | 1.06 | 0.18 |
| 13 | 1 | 15 | 5 | 2 | **0.5** | 0.3 | -0.2 | **0.3** | 0.3 | 0 | **-1.5** | -6.25 | -4.75 | **49.9** | 50.3 | 0.4 | **56.6** | 57.0 | 0.4 | **498** | 478 | -20 | **2.76** | 2.79 | 0.03 | **5.01** | 5.38 | 0.37 | **0.87** | 1.3 | 0.43 |
| 21 | 1 | 16 | 5 | 2 | **0.3** | 0.1 | -0.2 | **0.3** | 0.1 | -0.2 | **-1.25** | 0 | 1.25 | **47** | 46.8 | -0.2 | **60.2** | 58.5 | -1.7 | **540** | 460 | -80 | **2.51** | 2.33 | -0.18 | **3.77** | 3.83 | 0.06 | **1.47** | 1.74 | 0.27 |
| 22 | 1 | 16 | 5 | 1 | **1.3** | 0.5 | -0.8 | **0.5** | 0.5 | 0 | **-4.5** | -7.5 | -3 | **52.8** | 51.7 | -1.1 | **62.8** | 63.3 | 0.5 | **469** | 462 | -7 | **5.04** | 3.5 | -1.54 | **6.73** | 5.81 | -0.92 | **1.53** | 1.7 | 0.17 |
| 31 | 2 | 17 | 5 | 1 | **0.4** | 0.1 | -0.3 | **0.4** | 0.1 | -0.3 | **-3.625** | -1.5 | 2.125 | **45.4** | 45.2 | -0.2 | **57.1** | 58.6 | 1.5 | **450** | 437 | -13 | **2.62** | 2.58 | -0.04 | **4.51** | 3.89 | -0.62 | **1.99** | 2.25 | 0.26 |
| 31 | 2 | 17 | 5 | 2 | **0.2** | -0.1 | -0.3 | **0** | -0.2 | -0.2 | **-3.125** | -0.875 | 2.25 | **42.9** | 42.7 | -0.2 | **47.2** | 48.3 | 1.1 | **472** | 456 | -16 | **0.12** | 0.48 | 0.36 | **0.99** | 1.52 | 0.53 | **1.43** | 1.85 | 0.42 |
| 3 | 2 | 6 | 5 | 1 | **0.8** | 0.7 | -0.1 | **0.2** | 0.1 | -0.1 | **-7** | -7 | 0 | **46.3** | 45.8 | -0.5 | **49.3** | 47.6 | -1.7 | **570** | 564 | -6 | **1.15** | 0.81 | -0.34 | **1.16** | 1.57 | 0.41 | **0** | 0 | 0 |
| 3 | 2 | 6 | 5 | 2 | **0.5** | 0.5 | 0 | **0.2** | 0.1 | -0.1 | **-5.25** | -5.5 | -0.25 | **46.4** | 46 | -0.4 | **49.9** | 48.7 | -1.2 | **574** | 558 | -16 | **1.26** | 0.64 | -0.62 | **1.47** | 1.7 | 0.23 | **0** | 0.02 | 0.02 |
| 14 | 2 | 15 | 5 | 1 | **0.8** | 0.3 | -0.5 | **0.4** | 0.2 | -0.2 | **-3.625** | -2.75 | 0.875 | **45.8** | 46.1 | 0.3 | **51.8** | 52.4 | 0.6 | **493** | 502 | 9 | **2.1** | 2.18 | 0.08 | **2.95** | 2.87 | -0.08 | **0.95** | 0.82 | -0.13 |
| 150 | 2 | 32 | 5 | 2 | **1.4** | 1.2 | -0.2 | **0.2** | 0.2 | 0 | **-6.5** | -6.875 | -0.375 | **45.7** | 42.6 | -3.1 | **50.4** | 50.2 | -0.2 | **458** | 554 | 96 | **2.02** | 0 | -2.02 | **2.97** | 0 | -2.97 | **1.81** | 1.08 | -0.73 |
| 179 | 2 | 31 | 1 | 1 | **0.8** | 0.7 | -0.1 | **0.20** | 0.00 | -0.20 | **-7.250** | -4.750 | 2.500 | **49.1** | 49.9 | 0.8 | **60.2** | 60.7 | 0.5 | **417** | 417 | 0 | **2.99** | 3.06 | 0.07 | **7** | 6.35 | -0.65 | **2.65** | 2.66 | 0.01 |
| 179 | 2 | 31 | 1 | 2 | **0.8** | 0.9 | 0.1 | **0.30** | 0.10 | -0.20 | **-8.125** | -7.500 | 0.625 | **52.6** | 53.1 | 0.5 | **65.1** | 65.6 | 0.5 | **403** | 405 | 2 | **4.72** | 5.13 | 0.41 | **7.81** | 7.25 | -0.56 | **2.93** | 2.9 | -0.03 |
| 180 | 1 | 15 | 1 | 2 | **0.7** | 0.5 | -0.2 | **0.30** | 0.40 | 0.10 | **-2.250** | -4.500 | -2.250 | **62.6** | 61.9 | -0.7 | **83.4** | 83.8 | 0.4 | **384** | 376 | -8 | **11** | 10.77 | -0.23 | **11.76** | 11.87 | 0.11 | **3.16** | 3.24 | 0.08 |
| 194 | 2 | 28 | 1 | 2 | **0.5** | 0.3 | -0.2 | **0.2** | 0 | -0.20 | **-5.75** | -2.25 | 3.500 | **47.9** | 48.6 | 0.7 | **58.1** | 59.4 | 1.3 | **453** | 436 | -17 | **2.66** | 2.73 | 0.07 | **4.65** | 4.42 | -0.23 | **1.93** | 2.27 | 0.34 |
| 195 | 2 | 28 | 1 | 2 | **0.5** | 0.3 | -0.2 | **0.3** | 0.2 | -0.10 | **-3.75** | -1 | 2.750 | **49** | 49.3 | 0.3 | **59.3** | 59.6 | 0.3 | **427** | 434 | 7 | **2.77** | 2.71 | -0.06 | **4.92** | 4.94 | 0.02 | **2.45** | 2.32 | -0.13 |
| 180 | 1 | 15 | 2 | 1 | **0.70** | 0.70 | 0.00 | **0.20** | 0.30 | 0.10 | **-2.125** | -5.750 | -3.625 | **52.4** | 52.9 | 0.5 | **65.5** | 63.3 | -2.2 | **418** | 416 | -2 | **5.77** | 5.95 | 0.18 | **7.8** | 7.27 | -0.53 | **2.64** | 2.68 | 0.04 |
| 181 | 2 | 19 | 2 | 2 | **0.90** | 0.50 | -0.40 | **0.40** | 0.30 | -0.10 | **-2.625** | 1.000 | 3.625 | **51.5** | 52.2 | 0.7 | **64.6** | 64.8 | 0.2 | **419** | 420 | 1 | **3.25** | 3.39 | 0.14 | **5.82** | 5.81 | -0.01 | **2.62** | 2.6 | -0.02 |
| 187 | 1 | 19 | 5 | 1 | **1.2** | 0.80 | -0.40 | **0** | 0.00 | 0.00 | **-4.125** | -5.25 | -1.125 | **43** | 43 | 0 | **45.8** | 44.9 | -0.9 | **515** | 510 | -5 | **0** | 0 | 0 | **1.05** | 1.93 | 0.88 | **0.64** | 0.71 | 0.07 |
| 188 | 1 | 24 | 5 | 2 | **1** | 0.90 | -0.10 | **0.7** | 0.40 | -0.30 | **-12.75** | -10.75 | 2.000 | **49.4** | 48.6 | -0.8 | **61.5** | 56 | -5.5 | **487** | 453 | -34 | **3.46** | 2.86 | -0.6 | **4.63** | 5.77 | 1.14 | **1.08** | 1.93 | 0.85 |
| 182 | 1 | 20 | 5 | 2 | **1.3** | 1.3 | 0.00 | **0.7** | 1 | 0.30 | **-13.625** | -5 | 8.625 | **51.3** | 53.8 | 2.5 | **69.1** | 68.1 | -1 | **487** | 491 | 4 | **2.55** | 2.28 | -0.27 | **3.82** | 4.85 | 1.03 | **1.07** | 0.98 | -0.09 |
| 183 | 1 | 17 | 5 | 2 | **1.4** | 0.9 | -0.50 | **0.3** | 0.1 | -0.20 | **-6.25** | -6.375 | -0.125 | **44.4** | 44.3 | -0.1 | **46.1** | 45.6 | -0.5 | **476** | 465 | -11 | **0.06** | 0 | -0.06 | **0** | 0 | 0 | **1.34** | 1.61 | 0.27 |
| 184 | 1 | 15 | 5 | 2 | **0.2** | 0.3 | 0.10 | **0.2** | 0.3 | 0.10 | **-4.5** | -2.375 | 2.125 | **47.5** | 46.8 | -0.7 | **58.5** | 57.2 | -1.3 | **472** | 452 | -20 | **2.57** | 2.25 | -0.32 | **4.62** | 5.11 | 0.49 | **1.44** | 1.95 | 0.51 |
| 185 | 1 | 16 | 5 | 2 | **0.9** | 0.6 | -0.30 | **0.4** | 0.5 | 0.10 | **-10.5** | -7 | 3.500 | **51.9** | 49.8 | -2.1 | **66.5** | 60.5 | -6 | **464** | 433 | -31 | **4.63** | 3.38 | -1.25 | **6.48** | 7 | 0.52 | **1.65** | 2.34 | 0.69 |
| 186 | 1 | 17 | 5 | 2 | **0.4** | 0.2 | -0.20 | **0.3** | 0 | -0.30 | **-3.5** | -3.5 | 0.000 | **44.5** | 44.5 | 0 | **51.1** | 50.5 | -0.6 | **473** | 466 | -7 | **0.93** | 0.71 | -0.22 | **2.7** | 2.94 | 0.24 | **1.42** | 1.6 | 0.18 |
| 194 | 2 | 28 | 5 | 2 | **0.9** | 0.9 | 0.00 | **0.5** | 0.5 | 0.00 | **-7.75** | -4 | 3.750 | **45.8** | 44.1 | -1.7 | **50.9** | 48.2 | -2.7 | **474** | 444 | -30 | **1.26** | 0.01 | -1.25 | **2.54** | 2.7 | 0.16 | **1.4** | 2.12 | 0.72 |
| 182 | 1 | 20 | 6 | 1 | **0.7** | 0.7 | 0.00 | 0.2 | 0 | -0.20 | **-5.5** | -3 | 2.500 | **44.3** | 44.2 | -0.1 | **49** | 48 | -1.0 | **505** | 518 | 13 | **0.98** | 0.76 | -0.22 | **2.44** | 2.41 | -0.03 | **0.78** | 0.59 | -0.19 |
| 183 | 1 | 17 | 6 | 1 | **1.1** | 1.2 | 0.10 | 0 | 0 | 0.00 | **-7.5** | -8 | -0.500 | **44.5** | 44.6 | 0.1 | **45.2** | 45.3 | 0.1 | **474** | 473 | -1 | **0.2** | 0.26 | 0.06 | **0** | 0 | 0 | **1.39** | 1.41 | 0.02 |
| 186 | 1 | 17 | 6 | 1 | **0.5** | 0.4 | -0.10 | 0.5 | 0.3 | -0.20 | **-3.25** | -6.75 | -3.500 | **48.6** | 49.2 | 0.6 | **70.7** | 66 | -4.7 | **449** | 465 | 16 | **4.97** | 4.13 | -0.84 | **7.29** | 6.25 | -1.04 | **2.02** | 1.62 | -0.4 |
| 181 | 2 | 19 | 6 | 1 | **0.2** | 0.2 | 0.00 | 0 | 0.1 | 0.10 | **-0.5** | -0.75 | -0.250 | **43.5** | 43.1 | -0.4 | **45** | 44.2 | -0.8 | **478** | 481 | 3 | **0** | 0 | 0 | **0** | 0 | 0 | **1.28** | 1.23 | -0.05 |
| 189 | 1 | 27 | 6 | 1 | **0** | -0.1 | -0.10 | 0 | -0.1 | -0.10 | **0** | 0 | 0.000 | **43.5** | 43.6 | 0.1 | **45.1** | 45.4 | 0.3 | **539** | 551 | 12 | **0.04** | 0 | -0.04 | **2.09** | 1.37 | -0.72 | **0.29** | 0.12 | -0.17 |
| 191 | 1 | 15 | 6 | 1 | **1.1** | 1 | -0.10 | 0.3 | 0.2 | -0.10 | **-7** | -6.125 | 0.875 | **47.6** | 47.3 | -0.3 | **57.2** | 56.7 | -0.5 | **459** | 460 | 1 | **2.44** | 2.44 | 0 | **3** | 2.85 | -0.15 | **1.77** | 1.73 | -0.04 |
| 191 | 1 | 15 | 6 | 2 | **1.7** | 1.1 | -0.60 | 0.1 | 0 | -0.10 | **-8.125** | -7.875 | 0.250 | **45.2** | 45 | -0.2 | **47.7** | 47 | -0.7 | **478** | 474 | -4 | **0.44** | 0.44 | 0 | **0.78** | 0.28 | -0.5 | **1.31** | 1.41 | 0.1 |
| 192 | 1 | 17 | 6 | 2 | **0.5** | 0.5 | 0.00 | 0.1 | 0.2 | 0.10 | **-6.75** | -6 | 0.750 | **48.8** | 49.1 | 0.3 | **53.9** | 53.6 | -0.3 | **506** | 510 | 4 | **2.65** | 2.62 | -0.03 | **4.67** | 4.94 | 0.27 | **0.77** | 0.71 | -0.06 |
| 190 | 1 | 14 | 6 | 2 | **0.8** | 0.6 | -0.20 | 0.5 | 0.4 | -0.10 | **-5.375** | -9.25 | -3.875 | **45.9** | 47.6 | 1.7 | **52.5** | 57.1 | 4.6 | **507** | 504 | -3 | **2.29** | 2.81 | 0.52 | **3.09** | 4.28 | 1.19 | **0.75** | 0.79 | 0.04 |
| 187 | 1 | 19 | 7 | 2 | **1.2** | 1.1 | -0.1 | 0.2 | 0.3 | 0.1 | **-4.5** | -5 | -0.5 | **45.5** | 44.9 | -0.6 | **50.8** | 50.2 | -0.6 | **503** | 495 | -8 | **2.27** | 2.04 | -0.23 | **3.25** | 3.89 | 0.64 | **0.82** | 0.93 | 0.11 |
| 193 | 1 | 16 | 6 | 1 | **1.3** | 0.5 | -0.80 | 0.5 | 0.5 | 0.00 | **-4.5** | -7.5 | -3.000 | **52.8** | 51.7 | -1.1 | **63.8** | 61.3 | -2.5 | **469** | 462 | -7 | **5.04** | 3.5 | -1.54 | **6.73** | 5.81 | -0.92 | **1.53** | 1.7 | 0.17 |
